# Integrative epigenomic and genomic filtering for methylation markers in hepatocellular carcinomas

**DOI:** 10.1186/s12920-015-0105-1

**Published:** 2015-06-10

**Authors:** Jing Shen, Clare LeFave, Iryna Sirosh, Abby B. Siegel, Benjamin Tycko, Regina M. Santella

**Affiliations:** Department of Environmental Health Sciences, Mailman School of Public Health, Columbia University Medical Center, New York, NY 10032 USA; Institute for Cancer Genetics, Herbert Irving Comprehensive Cancer Center, Columbia University Medical Center, New York, NY 10032 USA; Department of Medicine, Columbia University Medical Center, New York, NY 10032 USA; Department of Pathology and Cell Biology, Columbia University College of Physicians and Surgeons, New York, NY 10032 USA

## Abstract

**Background:**

Epigenome-wide studies in hepatocellular carcinoma (HCC) have identified numerous genes with aberrant DNA methylation. However, methods for triaging functional candidate genes as useful biomarkers for epidemiological study have not yet been developed.

**Methods:**

We conducted targeted next-generation bisulfite sequencing (bis-seq) to investigate associations of DNA methylation and mRNA expression in HCC. Integrative analyses of epigenetic profiles with DNA copy number analysis were used to pinpoint functional genes regulated mainly by altered DNA methylation.

**Results:**

Significant differences between HCC tumor and adjacent non-tumor tissue were observed for 28 bis-seq amplicons, with methylation differences varying from 12% to 43%. Available mRNA expression data in Oncomine were evaluated. Two candidate genes (*GRASP* and *TSPYL5*) were significantly under-expressed in HCC tumors in comparison with precursor and normal liver tissues. The expression levels in tumor tissues were, respectively, 1.828 and − 0.148, significantly lower than those in both precursor and normal liver tissue. Validations in an additional 42 paired tissues showed consistent under-expression in tumor tissue for *GRASP* (−7.49) and *TSPYL5* (−9.71). A highly consistent DNA hypermethylation and mRNA repression pattern was obtained for both *GRASP* (69%) and *TSPYL5* (73%), suggesting that their biological function is regulated by DNA methylation. Another two genes (*RGS17* and *NR2E1*) at Chr6q showed significantly decreased DNA methylation in tumors with loss of DNA copy number compared to those without, suggesting alternative roles of DNA copy number losses and hypermethylation in the regulation of *RGS17* and *NR2E1*.

**Conclusions:**

These results suggest that integrative analyses of epigenomic and genomic data provide an efficient way to filter functional biomarkers for future epidemiological studies in human cancers.

**Electronic supplementary material:**

The online version of this article (doi:10.1186/s12920-015-0105-1) contains supplementary material, which is available to authorized users.

## Background

Epigenome-wide association studies [1–7], including ours [8, 9] have identified large panels of genes with aberrant DNA methylation in hepatocellular carcinoma (HCC). In two previous studies, we found an overlap of 402 significantly hypermethylated and 985 hypomethylated genes in HCC tumor tissues in comparison with adjacent non-tumors using Illumina 27K and 450K methylation arrays [8, 9]. Hypermethylation of 275 of the genes was consistent with other epigenome-wide studies [1–7], and the gene list includes several well-known tumor suppressor genes (TSGs) such as *APC* (adenomatous polyposis coli), *p16/CDKN2A* (cyclin-dependent kinase inhibitor 2A) and *RASSF1* (Ras association domain family member 1). These data indicate the reliability of genome-wide methylation results. Simultaneously, a large number of genes were identified for the first time as aberrantly methylated (127 hypermethylated and 308 hypomethylated) in HCC tumor tissue [8, 9] providing a resource to examine novel etiological risk factors and biologically relevant epigenetic markers for early diagnosis of HCC. Whether the aberrant methylation has functional consequences and can serve as sensitive markers of HCC is largely unknown in population-based epidemiological studies. Moreover, there is a lack of systematic analyses that integrate DNA methylation changes with genetic/epigenetic factors (copy number variations (CNVs), microRNA (miRNA) expression and histone modification, etc.) that potentially influence a gene’s biological functions. From the viewpoint of epidemiological studies alone, evidence of significant differences in DNA methylation between tumor and non-tumor tissues is insufficient to establish a causative role for the candidate genes in tumorigenesis. The large number of genes identified in previous epigenome-wide association studies [1–9] has complicated their application in larger population-based validation studies to determine in a cost-effective way their utility as risk factors, as well as early diagnostic/prognostic markers. Therefore, comprehensive analyses of available genetic and epigenetic data together may help us to better understand the functions of genes identified as being mainly regulated by aberrant DNA methylation, and narrow down the number of crucial methylation markers involved in hepatocarcinogenesis for future large epidemiological studies, including those which might involve using blood as an indirect surrogate, or cell-free tumor-derived DNA as a directly relevant analyte.

A limited number of CpG sites per gene or region are analyzed in genome-wide array-based methylation studies. Thus, we conducted further analysis by targeted next-generation bisulfite sequencing (bis-seq) to validate the genes identified as having the largest changes in DNA methylation based on both Illumina 27K and 450K array data. Our targeted bis-seq approach used PCR based amplification followed by sequencing on an Illumina MiSeq to cover multiple CpG sites (2–57 CpG sites) in candidate genes. This method allows for nucleotide level resolution and high sequencing depth and was able to cover multiple CpG sites for each of the candidate genes. To further investigate genes of interest and identify genes with inverse associations between DNA methylation and expression, we integrated the DNA methylation data with a genomic database of mRNA expression in HCC tissues and other cancer tissues, as well as cancer cell lines. Finally, we further examined the influence of genetic/epigenetic factors (such as CNVs, miRNA expression, histone modifications, etc) on gene expression to distinguish functional candidates mainly regulated by DNA methylation that may serve as promising early diagnostic markers.

## Methods

### Study subjects

This study was approved by the Institutional Review Board of Columbia University Medical Center. ). A waiver of consent was given because the majority of patients died before the research was carried out. Some of the living patients did give informed consent due to their interests in participating in further follow-up study. Sixty-six frozen HCC tissues from the Molecular Pathology Shared Resource of the Herbert Irving Comprehensive Cancer Center, as well as their detailed histological and clinical features including HBV (HBsAg) and HCV (anti-HCV) status were available from our prior study of methylation using Illumina arrays [9]. Twenty-four of these paired tumor/adjacent non-tumor tissues with different viral status were selected for targeted bis-seq of multiple CpG sites to determine methylation level, as well as for mRNA expression measured by quantitative reverse transcription PCR (qRT-PCR).

### Selection of candidate genes and regions

To select candidate genes and regions for further validation of DNA methylation by targeted bis-seq, we compared the significant CpG sites and genes identified in our previous studies using Illumina 450K and 27K arrays that were deposited in NCBI’s Gene Expression Omnibus (GEO) database (accession number GSE54751 and GSE37988) [8, 9]. We found an overlap of 402 hypermethylated genes (covering 505 CpG sites) and 985 hypomethylated genes (covering 1242 CpG sites). Because previous studies using a candidate gene approach have found more hypermethylated TSGs and DNA repair genes [10–21] than hypomethylated oncogenes in HCC, we selected a total of 20 hypermethylated and eight hypomethylated genes for the current study. Each gene had a >20% DNA methylation difference between HCC tumor and non-tumor tissues and a Bonferroni adjusted *p* value <0.05. For each hypermethylated gene, at least two amplicons were designed to cover candidate CpG sites, and one amplicon was designed for hypomethylated genes. Finally, a total of 48 PCR primers pairs were used, and after sequencing, low quality sequences were removed and genome alignment carried out. Twenty-nine of the amplicons covering 20 genes were successful and analyzed in the current study.

### Laboratory methods

Bisulfite treatment was performed on 1μg DNA using the Epitect kit (Qiagen) as per the instructions. The genomic locations of candidate genes and covered CpG sites of the amplicons for targeted bis-seq are given in Additional file 1: Table S1. Oligonucleotide primers were designed around the CpGs of interest using MethPrimer (http://www.urogene.org/cgi-bin/methprimer/methprimer.cgi) [22], and the CS1 and CS2 Fluidigm tags were added. Primers were synthesized (IDTDNA) and verification of the amplicon size was performed. The Fluidigm Access Array was performed with the KAPA HiFi 2x Uracil + polymerase. Next the adapter and barcode sequences were added following the Fluidigm protocol using the Faststart Hi Fidelity kit (Roche) and the barcoded primers (Fluidigm). Verification of product was done on an agarose gel and cleaned-up using the Agencourt AMPure XP (Beckman Coulter). Libraries were then quantified using the Kapa Library Quantification Kit (Kapabiosystems). Samples were then pooled with 30–50% PhiX (Illumina) and loaded onto the MiSeq (Illumina) for sequencing. Libraries are clustered and sequenced with 250 nucleotide paired-ends. The Fastq files generated by sequencing were trimmed for both adapters and for a quality cut off of 30 using Trim Galore (http://www.bioinformatics.babraham.ac.uk/projects/trim_galore/). Sequencing alignment and methylation calls were done via Bismark [23] and bowtie2 [24]. The genome used for alignment was recent human assembly GRCh37/hg19.

Total RNA, including miRNA was isolated from frozen HCC tumor and adjacent non-tumor tissues by Qiazol and RNeasy Microarray Tissue Mini Kits (Qiagen) according to the manufacturer’s protocol. For mRNA expression, 1.0 μg isolated RNA (10μL) was converted to cDNA using the High-Capacity cDNA Reverse Transcription Kit. After 10 times dilution of the RT products, TaqMan® Gene Expression Assays (Life Technologies) were used to detect two candidate genes *GRASP* (general receptor for phosphoinositides 1-associated scaffold protein: Hs00699132_g1) and *TSPYL5* (TSPY-like 5: Hs00603217_s1). Data were normalized by the housekeeping gene *GAPDH* (glyceraldehyde-3-phosphate dehydrogenase: Hs02758991_g1) as recommended by a study to validate putative reference genes in HCC tissues [25]. TaqMan Low Density Arrays (TLDA, Life Technologies), covering 670 unique human mature miRNAs were used to generate genome-wide miRNA profiles that was deposited in NCBI’s GEO database (accession number GSE54751) [26].

### Integrative analyses

The Oncomine database (https://www.oncomine.org) [27, 28] that includes cancer microarray data deposited in GEO and the Stanford Microarray Database (SMD) were used to determine the differences in mRNA expression between HCC tumor and/or precursor/normal liver tissues for 20 candidate genes, as well as the well-known TSGs *APC, CDKN2A* and *RASSF1* [8, 9]. Fifteen publicly available datasets were selected for the integrative analyses (http://tcga-data.nci.nih.gov/tcga/) [29–42]. Details of standardized normalization techniques and statistical methods can be found on the Oncomine website [27, 28]. The gene expression data were log_2_ transformed, median centered per array, and the standard deviation (SD) was normalized to one per array [27, 28]. Genes with significant differences in mRNA expression (*p* ≤ 0.05) and a concordant DNA methylation and mRNA expression pattern (i.e. hypermethylation with under-expression or hypomethylation with up-regulation) in liver tissues were further examined for the potential impacts of CNVs (gain or loss) using both Oncomine and our 450K methylation intensity data by DNA-Chip Analyzer (dChip) [43]. Others were excluded from further analysis. The expression levels of miRNAs (increase or decrease) that target hypermethylated genes without losses of CNVs were analyzed. The target genes of miRNAs were identified from an online resource (http://c1.accurascience.com/miRecords/). Finally, Encyclopedia of DNA Elements (ENCODE) data (https://genome.ucsc.edu/ ENCODE/) for an HCC cell line (HepG2) and another seven cancer cell lines (GM12878, H1-hESC, HSMM, HUVEC, K562, NHEK and NHLF) were incorporated with DNA methylation results from candidate genes to examine the co-operative role of histone modifications and deoxyribonuclease (DNase I) hypersensitivity on chromatin activity. Studies showed that active histone marks include histone 3 lysine 4 monomethylation (H3K4me1) typically associated with transcriptional enhancers; histone 3 lysine 4 trimethylation (H3K4me3) typically associated with promoters; and histone 3 lysine 27 acetylation (H3K27ac) typically associated with both active promoters and enhancers [44, 45]. Histone 3 lysine 27 trimethylation (H3K27me3) acts as a repressive histone marker to epigenetically control gene transcription [46]. DNase I sensitivity is an indicator of open chromatin, and DNase I hypersensitivity sites are typical marks for active regulatory regions [47].

### Statistical analysis

Paired t-tests with Bonferroni correction for multiple testing were used to compare differentially methylated genes between tumor and adjacent non-tumor tissues. A significant difference was defined as an amplicon with a Bonferroni-corrected *p*-value ≤ 0.05. Hierarchical clustering of data was performed with the significant amplicons by tissue status (tumor *vs.* adjacent non-tumor). Each gene or miRNA’s expression was separately assessed for tissue differences by one-sided Student’s t test (under-expression for hypermethylated genes or over-expression for hypomethylated genes). Pearson’s correlation was used to analyze the relationship between DNA methylation and mRNA/miRNA expression. Chi-square test was used to analyze the impacts of CNVs (gain, no change and loss) on gene expression (under- or up-regulation). Statistical analyses were conducted using Statistical Analysis System 9.0 (SAS Institute).

## Results and discussion

### Clinical and pathological characteristics of HCC patients

Clinical and pathological characteristics are described in Additional file 1: Table S2. The average age at HCC diagnosis is 57.1 ± 7.5 years. More patients are male (83%), Caucasian (54%) and positive for either HBV (33.3%) or HCV (33.3%) or both (16.7%). The same proportion (45.8%) is ever smokers or alcohol drinkers. Among HCC patients, 87.5% have pathologically defined cirrhosis and 62.5% have tumors grade III or IV.

### Comparison of DNA methylation results from targeted bis-seq and Illumina arrays

A total of 29 CpG amplicons covering 20 genes (15 hyper- and 5 hypo-methylated genes) were sequenced by targeted bis-seq. The average covered CpG sites for each gene was 22, and ranged from 2 to 57 (Table 1). Statistically significant methylation differences between tumor and non-tumor tissues (Bonferroni corrected *p* < 0.05) were observed for 28 amplicons with methylation differences varying from 12% to 43%, including 14 hyper- and 5 hypo-methylated genes. Only one hypermethylated gene (*DUOX1*) showed a non-significant tumor/non-tumor difference (*p* = 0.277). This suggests that about 5% (1/20) of genes identified by Illumina 450K array may be false positive findings even after adjustment for multiple comparison and using stringent selection criteria.

**Table 1:** Targeted bis-seq data for methylation in 20 candidate genes (29 amplicons) in 24 paired HCC tumor and adjacent non-tumor tissues

Gene symbol	Amplicons name	No. of covered CpG sites	Non-tumor	Tumor	Difference	Adjusted *P* value *
			Mean, SD	Mean, SD		
*CDKL2*	CDKL2.a	11	6.49 (8.35)	33.52 (18.80)	27.03	2.50E-04
*CDKL2*	CDKL2.b	15	7.59 (11.57)	41.29 (21.98)	33.70	1.94E-04
*CDKL2*	CDKL2.c	27	13.20 (13.50)	37.15 (20.68)	23.95	2.83E-02
*CLCN1*	CLCN1	28	16.31 (10.83)	47.84 (21.93)	31.53	3.01E-04
*DUOX1*	DUOX1	36	16.93 (16.93)	36.98 (24.43)	20.05	2.77E-01
*GRASP*	GRASP.a	24	12.66 (8.70)	42.45 (23.56)	29.79	3.92E-04
*ILDR2*	ILDR2.a	23	7.66 (4.22)	43.18 (27.32)	35.52	4.21E-05
*MAST1*	MAST1.a	16	16.40 (20.56)	59.15 (33.23)	42.75	4.99E-04
*MAST1*	MAST1.b	2	10.42 (10.98)	34.74 (21.02)	24.32	4.33E-03
*MAST1*	MAST1.c	19	15.25 (8.44)	38.92 (14.03)	23.67	5.15E-05
*NKX6-2*	NKX6-2.b	24	26.17 (9.56)	54.97 (17.70)	28.80	4.25E-05
*OTX1*	OTX1.a	8	22.53 (9.14)	52.15 (24.09)	29.62	2.63E-03
*OTX1*	OTX1.b	28	17.68 (12.10)	49.60 (17.44)	31.92	1.85E-04
*SERHL*	SERHL.a	16	11.94 (4.38)	35.71 (13.19)	23.77	3.82E-07
*SERHL*	SERHL.b	11	4.86 (2.26)	17.15 (12.24)	12.29	1.08E-03
*SPAG6*	SPAG6.b	53	16.07 (5.05)	34.12 (15.67)	18.04	1.59E-03
*SPDYA*	SPDYA.b	11	46.93 (17.40)	73.91 (18.14)	26.98	1.29E-02
*TRIL*	TRIL.a	16	18.13 (6.02)	53.25 (20.58)	35.12	1.01E-05
*TRIL*	TRIL.b	22	13.88 (8.85)	36.95 (17.75)	23.07	2.22E-03
*TRIL*	TRIL.c	17	9.73 (6.70)	41.44 (17.52)	31.71	6.62E-06
*TRIL*	TRIL.e	44	12.10 (10.22)	38.52 (19.07)	26.42	1.55E-03
*TSPYL5*	TSPYL5	57	13.83 (12.12)	41.21 (18.79)	27.38	8.09E-04
*USP44*	USP44.c	9	61.31 (10.17)	73.45 (10.01)	12.14	6.81E-03
*ZNF397OS*	ZNF397OS.a	32	13.76 (11.59)	38.94 (18.21)	25.18	3.10E-04
*FAM66B*	FAM66B	13	91.18 (3.59)	71.89 (17.30)	−19.29	9.49E-04
*KCNQ2*	KCNQ2	44	74.06 (14.65)	45.21 (22.68)	−28.85	3.99E-03
*PROKR2*	PROKR2	14	83.57 (14.68)	65.11 (12.97)	−18.46	1.11E-02
*PTPRN2*	PTPRN2	8	74.68 (11.71)	53.60 (17.39)	−21.08	6.50E-03
*REXO1L2P*	REXO1L2P	12	84.14 (14.58)	53.98 (22.99)	−30.16	1.75E-03

* All adjusted *p* values are less than 0.05 with Bonferroni correction for multiple testing except *DUOX1*

The results of targeted bis-seq are shown for *GRASP* (25 CpG sites) and *TSPYL5* (57 CpG sites) in Additional file S1: Figure S1. Generally, within each sample, DNA methylation levels across different CpG sites were consistent regardless of tumor status. For hypermethylated genes, most tumor tissues displayed higher levels of DNA methylation compared to adjacent non-tumor tissues for each individual CpG site and the mean of all CpG sites. The direction of the methylation difference (hyper- or hypo-) between tumor and non-tumor tissues for targeted bis-seq was 100% concordant with that from 27K and 450K data [8, 9]. Additional file 1: Figure S2 shows two examples of mean DNA methylation comparisons for *GRASP* and *TSPYL5* by targeted bis-seq and 450K array approaches. Statistically significant DNA hypermethylation was observed in HCC tumor tissue compared to non-tumor tissue for both genes. These data demonstrated the accuracy and reliability of both targeted bis-seq and Illumina 27K/450K methylation arrays.

### Comparison of mRNA expression in HCC tumor, precursors and normal liver tissues

With more and more epigenomic and genomic studies completed in tumor tissues, large panels of candidate genes are available that need further verification for their biological functions before they can be applied to population-based molecular epidemiological studies. Comparison of mRNA expression patterns is an effective way to identify relevant functional changes and focus on crucial methylation markers involved in tumorigenesis.

**Fig. 1:**
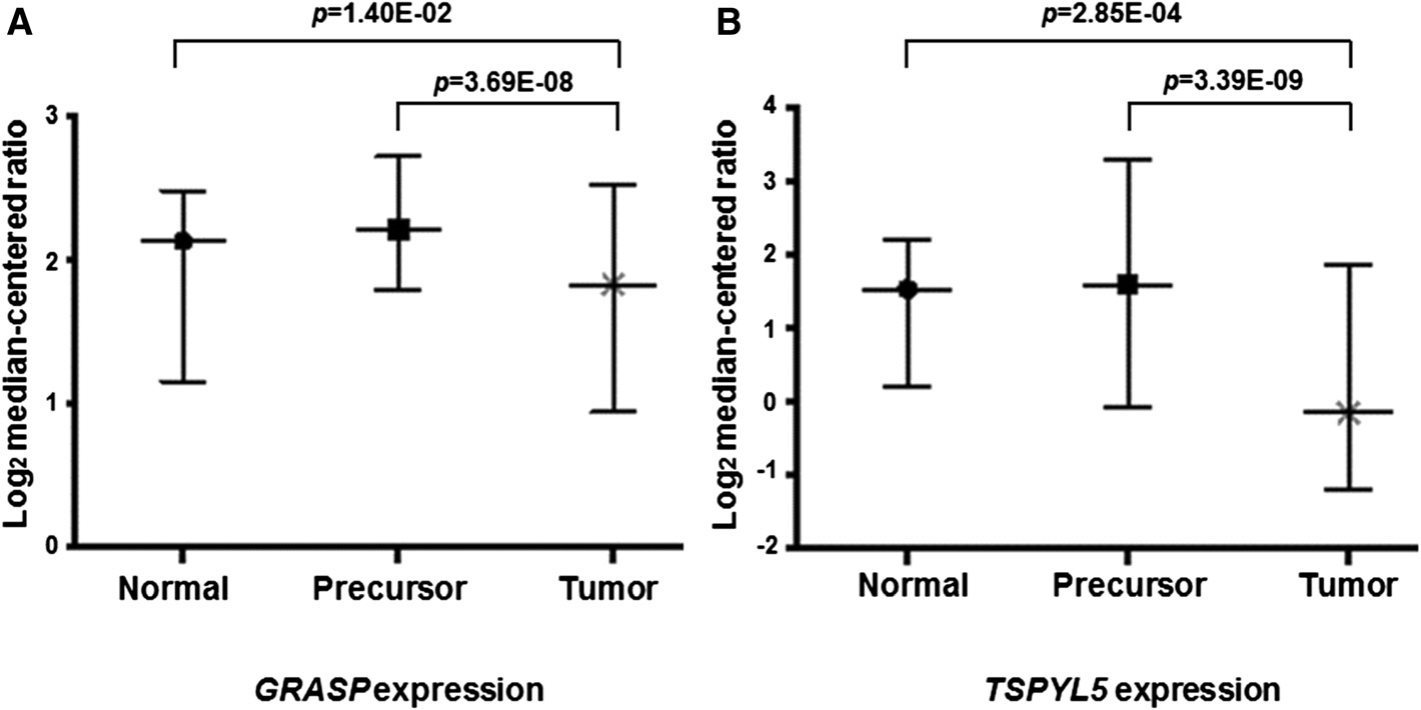
Comparisons of mRNA expression for *GRASP* and *TSPYL5* in HCC tumor, precursor and normal liver tissues. Log_2_ median-centered ratio represents the mRNA expression levels for *GRASP* (**a**) and *TSPYL5* (**b**) in HCC tumor, precursor and normal liver tissues. Statistically significant under-expression was observed in HCC tumor tissues for *GRASP* and *TSPYL5* compared to both precursor and normal liver tissues. The data were extracted from public Oncomine databases

A total of 15 candidate genes (*CDKL2*, *CLCN1*, *DUOX1*, *MAST1*, *NKX6-2*, *OTX1*, *SPAG6*, *SPDYA*, *TRIL*, *USP44*, *ZNF397OS*, *GRASP*, *TSPYL5*, *KCNQ2*, and *PTPRN2*) and three known TSGs (*APC*, *CDKN2A* and *RASSF1*) with available mRNA expression data in the Oncomine database (Additional file 1: Table S3) were evaluated for their biological functions in HCC tumor (*n* = 418), precursor (*n* = 142), and normal liver tissues (*n* = 325). Another five genes (*ILDR2*, *SERHL*, *FAM66B*, *PROKR2*, and *REXO1L2P*) were omitted from the analysis of mRNA expression due to the lack of Oncomine data. As expected, the expression levels of *APC* and *CDKN2A* were significantly down-regulated in HCC tumor tissues compared to both precursor and normal liver tissues (Additional file 1: Table S4) [29–32]. Overall, four candidate genes (*CLCN1*, *DUOX1*, *GRASP* and *TSPYL5*) were significantly under-expressed in HCC tumors in comparison with precursor and normal liver tissues [29–32], but inconsistent non-significant differences were also observed for *CLCN1* and *DUOX1* expression between HCC and precursor [29, 30, 33]. The expression levels of *GRASP* and *TSPYL5* in HCC tumor tissues were, respectively, 1.828 and −0.148, which were significantly lower (Fig. 1) than those in both precursor (2.210 and 1.585) and normal liver tissue (2.134 and 1.527) [29]. Because DNA methylation changes between HCC tumor and non-tumor tissues did not achieve statistical significance for *DUOX1* (Table 1), down-regulation of its mRNA in tumor tissue was unlikely due to DNA methylation changes and was thus excluded from further analysis. Hypermethylated *ZNF397OS* was only significantly down-regulated in HCC tumor compared to precursor tissues [32], but not compared to normal liver tissue. Similarly, hypomethylated *KCNQ2* displayed significant over-expression in tumor compared to precursor tissue [30]. Another four hypermethylated genes (*CDKL2*, *MAST1*, *SPAG6* and *TRIL*) exhibited significant under-expression in tumor compared to normal liver tissue [29–33], but not precursor tissues. Hypomethylated *PTPRN2* was significantly over-expressed in tumor tissue compared to normal liver tissue (Additional file 1: Table S4) [30]. The heterogeneous expression patterns of these genes in HCC tumor vs. precursor and normal liver tissues need further clarification. Four hypermethylated genes (*NKX6-2*, *OTX1*, *SPDYA* and *USP44*) did not show any significant difference in mRNA expression among tissues [29, 32], suggesting a minor biological role in hepatocarcinogenesis, and were excluded from further analysis. The lack of expression data on five genes (*ILDR2*, *SERHL*, *FAM66B*, *PROKR2*, and *REXO1L2P*) in HCC tissues may lead us to miss some important candidate genes; but it is unlikely to impact the functional genes identified in the current study.

### Integrative analyses of CNVs and miRNAs

Integrative analyses of relevant genetic and epigenetic changes may help us to further understand important regulators and potential mechanisms associated with the biological functions of the genes that we have identified. Gains or losses of copy numbers in DNA have been associated with relevant differences in expression of mRNAs (up- or down-regulation) [48] and play a similar biological role as DNA hypo- or hyper-methylation. Integrative analyses of CNVs was performed in HCC tumor (*n* = 209), precursor (*n* = 94) and normal liver tissues (*n* = 145) based on the Oncomine database (Additional file 1: Table S3), as well as in our 66 paired HCC tissues. We found no significant CNV losses for *CLCN1*, *GRASP* and *TSPYL5,* genes that displayed significant under-expression in HCC tumor compared to both precursor and normal liver tissues (Additional file 1: Figure S3). Similarly, no significant CNV losses were observed for *MAST1*, *SPAG6* and *TRIL*, genes that showed under-expression in HCC tissue compared to normal liver tissue (data not shown).

**Fig. 2:**
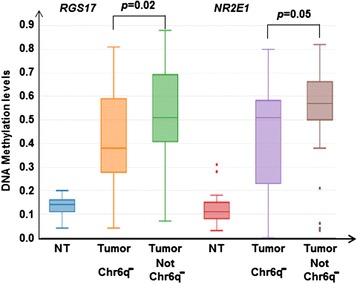
DNA methylation comparisons for HCC tumors with or without DNA copy number losses in Chr6q region. Chr6q^−^ indicates DNA copy number losses in this region, NT indicates non-tumor tissue. Two genes (*RGS17* and *NR2E1*) showed significantly decreased DNA methylation in HCC tumor tissues with DNA copy number losses in region Chr6q compared with tissues with no copy number losses

Integrating data on copy number losses and DNA methylation changes in HCC tumor tissues, we only found two CpG sites in *RGS17* (cg16924337) and *NR2E1* (cg17386213) that had significantly reduced DNA methylation in tumors with loss of DNA copy number compared to those without (Fig. 2). DNA methylation levels in tumor and non-tumor tissues were, respectively 0.54 and 0.41 for *RGS17* (*p* = 0.02), and 0.53 and 0.43 for *NR2E1* (*p* = 0.05), suggesting a potential regulatory role for copy number losses in liver tumorigenesis in addition to DNA methylation alteration. Therefore, one allele either undergoing DNA hypermethylation or loss of copy number may lead to the partial inactivation of genes with tumor suppressive activity.

Aberrant expression of miRNAs may also regulate function of the relevant target genes. Among the six hypermethylated and under-expressed genes (*CLCN1*, *GRASP*, *MAST1*, *SPAG6*, *TRIL* and *TSPYL5*) without losses of copy number, we further examined whether miRNAs that target those genes are over-expressed in HCC tumor tissue. The numbers of miRNAs targeting each gene varied from 4 to 51 and included 1-27 detectable miRNAs in liver tissue (Additional file 1: Table S5). Overall, no significant up-regulated miRNA was observed in HCC tumor tissue compared to non-tumor tissue. The expression of miR-888 targeting *SPAG6* did not show a significant difference (Log_2_ fold change = 0.11, *p* = 0.867). In contrast, we found 1-8 significantly under-expressed miRNAs (log_2_ fold changes ranging from −0.68 folds to −2.66 folds) in HCC tumors (Additional file 1: Table S6), suggesting those miRNAs were unlikely to cause the under-expression of the relevant target genes (*CLCN1*, *GRASP*, *MAST1*, *TRIL* and *TSPYL5*). These integrative analyses indicate that no significant CNV losses and over-expressed miRNAs that target *GRASP* and *TSPYL5* were observed in HCC (Table 2), suggesting a minor functional impact of CNVs and miRNAs for these two genes.

**Table 2:** Comparison of significantly expressed miRNAs that target *GRASP* and *TSPYL5*

Target gene symbol	miRNAs	Non-tumor	Tumor	Adjusted *P* value *	Regulation in HCC tumor
		Mean (Log2), SD	Mean (Log2), SD		
*GRASP*	hsa-miR-320b	−8.97, 0.75	−10.10, 0.96	8.95E-03	Down
*TSPYL5*	hsa-miR-193a-3p	−8.21, 1.06	−9.53, 0.88	7.58E-03	Down
	hsa-let-7g	−3.43, 0.67	−4.11, 0.55	2.32E-02	Down
	hsa-let-7a	−6.43, 0.64	−7.35, 0.86	1.42E-02	Down
	hsa-let-7e	−4.05, 0.82	−4.78, 0.63	3.86E-02	Down
	hsa-let-7c	−6.88, 0.50	−8.62, 1.08	2.24E-04	Down
	hsa-let-7b	−4.53, 0.86	−5.89, 1.18	8.94E-03	Down
	hsa-miR-320b	−8.97, 0.75	−10.10, 0.96	8.95E-03	Down
	hsa-miR-27b	−5.69, 0.79	−6.48, 0.70	2.84E-02	Down

* Bonferroni correction for multiple testing

### Validation of mRNAs expression for *GRASP* and *TSPYL5*

The two promising genes (*GRASP* and *TSPYL5*) were further validated in terms of their mRNA expression in the same 24 paired HCC tissues that underwent targeted bis-seq, as well as an additional 42 paired tissues. *GRASP* and *TSPYL5* were selected based on their consistent DNA methylation and expression patterns in tumor, precursor and normal tissues, as well as no significant influence of CNVs and miRNAs on their functions. The distribution of *GRASP* and *TSPYL5* expression levels for each sample are shown in Additional file 1: Figure S4. A consistent and statistically significant under-expression pattern was observed for *GRASP* and *TSPYL5* in both the 24 and 42 paired tumor tissues (Table 3). The fold-changes for *GRASP* and *TSPYL5* were, respectively −1.62 and −1.85 in the 24 pairs, and −1.96 and −1.71 in the 42 pairs, which is consistent with the Oncomine data (Additional file 1: Table S4). When analyzing correlation of DNA methylation and expression, we found consistent hypermethylation and repression pattern for *GRASP* (71% of samples) and *TSPYL5* (67%) in the 24 pairs (Additional file 1: Figure S5). In the additional 42 paired HCC tumor and adjacent non-tumor tissues, similar proportions of tumor tissues with a hypermethylation and under-expression pattern were observed for *GRASP* (68%) and *TSPYL5* (78%). The correlation coefficients of DNA methylation and expression were −0.394 for *GRASP* (*p* = 0.007) and -0.415 for *TSPYL5* (*p* = 0.004), indicating a major regulatory role for DNA methylation (Table 4). The repression of *GRASP* and *TSPYL5* observed in tumor tissue is more likely through the mechanism of altered DNA methylation.

**Table 3:** Log_2_ expression levels of two candidate mRNAs in discovery (24 pairs) and validation (42 pairs) sets by qRT-PCR assays

mRNAs	Samples	Log_2_ expression levels		Fold change	*P value*
		Mean (SD)			
		Tumor	Non-tumor		
*GRASP*	24 pairs	−7.21 (1.30)	−6.51 (0.89)	−1.62	2.75E-02
	42 pairs	−7.49 (1.41)	−6.52 (1.26)	−1.96	1.53E-03
*TSPYL5*	24 pairs	−9.67 (1.61)	−8.79 (1.13)	−1.85	3.68E-02
	42 pairs	−9.71 (1.63)	−8.94 (1.22)	−1.71	1.25E-02

**Table 4:** Integrative analyses for DNA methylation, mRNA expression and CNVs in HCC tumor tissues

Variables	*GRASP*	*TSPYL5*
DNA hypermethylation, No (%)	65 (98.5)	64 (97.0)
mRNA under-expression, No (%)	45 (69.2)	47 (73.4)
Expression difference, Mean (SD)	−1.63 (1.44)*	−1.67 (1.51)**
Methylation difference, Mean (SD)	0.55 (0.13)*	0.36 (0.11)**
Copy number loss, No (%)	4 (8.9)^†^	1 (2.1)^†^
Copy number gain, No (%)	14 (31.1)	33 (70.2)
mRNA over-expression, No (%)	20 (30.8)	17 (26.6)
Expression difference, Mean (SD)	0.93 (0.79)^#^	1.12 (1.38)^¶^
Methylation difference, Mean (SD)	0.44 (0.30)^#^	0.39 (0.07)^¶^
Copy number gain, No (%)	7 (35.0)^‡^	12 (70.6)^‡^
Copy number loss, No (%)	4 (30.0)	0 (0.0)

* Correlation coefficient = −0.394, *p* = 0.007; ** Correlation coefficient = −0.415, *p* = 0.004; ^†^ *p* = 0.153; ^#^ Correlation coefficient = −0.199, *p* = 0.387; ^¶^ Correlation coefficient = −0.032, *p* = 0.906; ^‡^ *p* = 0.031

### Integrative analyses with ENCODE data

**Fig. 3:**
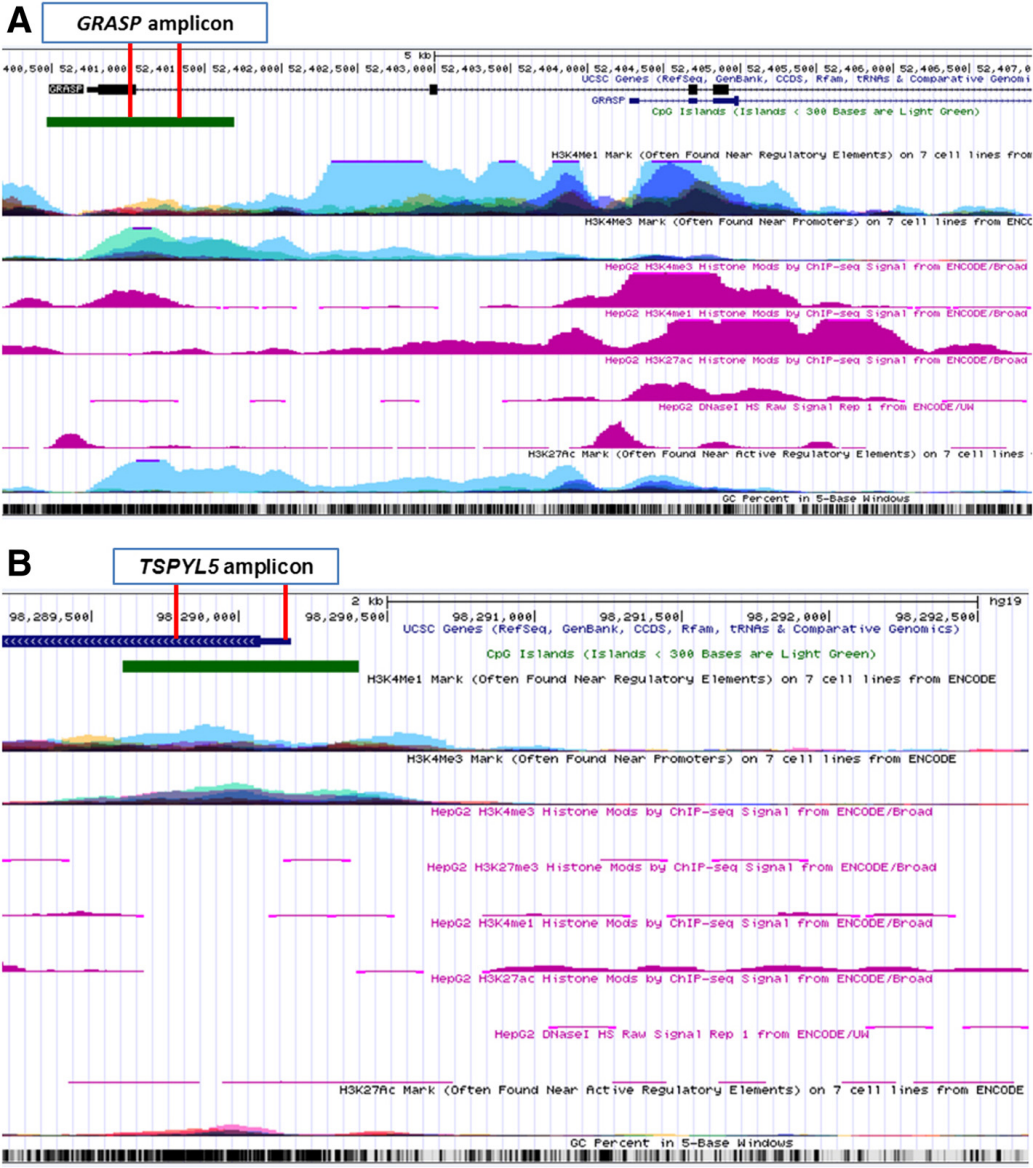
UCSC genome browser tracks showing histone modifications (H3K4me1, H3K4me3, H3K27me3 and H3K27ac) and DNase I cleavage states around *GRASP* (**a**) and *TSPYL5* (**b**) amplicons in HCC HepG2 and seven other cancer cell lines. Shown from top to bottom is the CpG island; layered H3K4Me1 activating mark, H3K4Me3 promoter specific mark in seven other cancer cell lines; H3K4Me3, H3K27me3, H3K4Me1, H3K27Ac activator marks and DNase I hypersensitive sites in HCC HepG2 cells; H3K27Ac activator in 7 other cancer cell lines; and GC percent. **a**: The genome browser map shows the genomic region around the *GRASP* (chr12:52,399,000-52,407,500). The bisulfite sequencing data covers 25 CpGs located within the promoter specific the 138^th^ CpG island as shown. Around the *GRASP* amplicon, no DNase I hypersensitivity peak was observed in HepG2 cells. Histone marks (H3K4me1 and H3K27ac) displayed no signature of active regulation in HepG2 cells, while a silencer of H3K27me3 showed a small hill. Unexpectedly, active histone H3K4me3 also exhibited a peak. The histone modifications for the seven other cancer cell lines displayed high peaks for H3K4me1, H3K4me3 and H3K27ac. **b**: The genome browser map shows the genomic region around *TSPYL5* (chr8:98,287,500-98,292,500). The bisulfite sequencing data covers 57 CpGs located within the promoter specific the 100^th^ CpG island as shown. Consistent with DNA hypermethylation and under-expression of mRNA, there were no active histone marks (H3K4me1, H3K4me3 and H3K27ac), as well as closed chromatin (no peak for DNase I hypersensitive sites) around the *TSPYL5* amplicon in HepG2 cells, which is different from the pattern observed in seven other cancer cell lines (showing high peaks for H3K4me1, H3K4me3 and H3K27ac marks). Unexpected, no activation of H3K27me3 was observed around *TSPLY5* in HepG2 cells

To perform integrative analyses of DNA methylation and the ENCODE data for *GRASP* and *TSPYL5* in HepG2 and seven other cancer cell lines, we examined the co-operative role of histone modifications and deoxyribonuclease (DNase I) hypersensitivity on chromatin activity. Around the *GRASP* amplicon located in the promoter region, no DNase I hypersensitivity peak was observed in HepG2 cells, indicating inactive chromatin (Fig. 3a). Further investigation of the *GRASP* promoter area showed that the active histone marks (H3K4me1 and H3K27ac) had no signature of up-regulation in HepG2, but H3K27me3, a repressor, displayed an increase which is consistent with the status of DNA hypermethylation in HCC tumor tissue. Similarly, neither DNase I hypersensitive sites, nor activation of histone marks (H3K4me1, H3K4me3 and H3K27ac) were observed around the *TSPYL5* amplicon in HepG2 cells (Fig. 3b). No increase of H3K27me3 was observed around *TSPLY5* in HepG2 cells. The different histone modifications in HepG2 and the other cell lines indicate a potentially specific role for *GRASP* and *TSPYL5* in hepatocarcinogenesis.

*GRASP* is located at chromosome 12q13.13 and encodes a 395 amino acid protein. Identified as an all-trans retinoic acid-induced gene [49], *GRASP* interacts with numerous neuronal proteins of cytohesin family members Grp1 and ADP-ribosylation factor (Art) [49] to play a role in the intracellular trafficking of receptors [50–52]. *GRASP* has been found to be significantly hypermethylated in HBV-infected HCC tumor tissue, but changes in expression were unknown [5]. Recently, *GRASP* was found to be significantly hypermethylated in colorectal cancer and also negatively correlated with expression levels [53]. The methylation level of *GRASP* is very low in non-neoplastic colorectal tissue and WBC DNA from healthy subjects, indicating potential usefulness as a non-invasive epigenetic markers [54]. It is known that *CDKN2A*^INK4a^ (a tumor suppressor gene) encodes p14ARF that acts as a checkpoint within the ARF-MDM2-p53 pathway to activate and stabilize p53 [55]. When a mutagenic event occurs, the expression of p14ARF can interrupt the abnormal cell proliferation [56], while the function of GRASP is to promote ARF-Rac signaling [57]. It is biologically plausible that methylation alterations in either *CDKN2A*^INK4a^ or *GRASP* may cause dysfunction of this pathway, and initiate tumorigenesis.

*TSPYL5* is a member of the testis-specific protein Y-encoded-like (TSPY-L) family of genes located on chromosome 8q22 and is a target of epigenetic silencing in gliomas including glioblastoma [58, 59] and gastric cancers [60]. Upon treatment with the demethylating agent 5-aza-dC in glioma [58] and gastric cancer [59] cell lines, TSPYL5 expression was restored, indicating epigenetic regulation. Stable transfection of *TSPYL5* in glioma or colon cancer cell lines inhibited growth [58, 60]. *TSPYL5* is frequently amplified in breast cancer, and displays an oncogene-like activity. The highest level of expression was found in basal-like breast cancers that correlated with shortened distant metastasis-free survival [61]. TSPYL5 can override senescence-like proliferation arrest and oncogene-induced senescence and contribute to cell transformation suggesting its role as a negative regulator of p53 function [61]. TSPYL5 protein was shown to interact with USP7 (a known deubiquitylating enzyme for p53), and suppress deubiquitination of p53 therefore marking it for degradation [61]. Moreover, the binding of TSPYL5 to USP7 can also effect other proteins targeted by USP7 for deubiquitylation including proteins involved in tumor necrosis factor alpha (TNF-α) induced apoptosis [62]. We observed decreased TSPYL5 levels which would lead to an increase in p53 protein levels. In normal cells, the p53 protein is low due to rapid degradation by the proteasome through MDM2 targeted ubiquitination. Protein levels of p53 have been shown to be higher in HCC relative to adjacent normal tissue [63] but in general p53 levels were variable among HCC samples [64]. Thus, *TSPYL5* hypermethylation could be a contributing factor to higher p53 protein levels in some HCC via its interaction with USP7. More studies are needed to determine the biological function of low TSPYL5 in HCC.

This integrative analyses and filtering for biologically important genes regulated by altered methylation is an important first step in the identification of biomarkers for epidemiologic studies. The novel findings of the current study are that the significant repression of *GRASP* and *TSPYL5* in HCC tumors is likely due to epigenetic regulation, while *RGS17* and *NR2E1* are functionally regulated by both DNA methylation and copy number losses. More importantly, many genes with aberrant DNA methylation were excluded from further evaluation because of lack of consistent evidence for their biological functions. Therefore, integrative analysis of available genetic and epigenetic data provides a high through-put and cost-effective tool to transform findings from the current study into the next step of population based epidemiological studies using non-invasive blood as a surrogate for target tissue. Integrative analysis also makes it feasible to robustly examine the tissue specificity and interpret results when evaluating the identified functional biomarkers in epidemiological studies. Because the ENCODE data does not include HCC cell lines (SNU-449, JHH2), we performed the integrative analyses on HepG2 cells derived from a human hepatoblastoma. The co-operation between DNA methylation and histone modifications on chromatin activity may be different for the different types of cell lines, and the results should be interpreted with caution.

## Conclusions

The integrative analyses of epigenomic and genomic profiles provide us with an effective tool to filter biologically functional epigenetic markers for future epidemiological studies. For the first time, the expression of *GRASP* and *TSPYL5* were identified mainly regulated by DNA methylation, while *RGS17* and *NR2E1* genes may be repressed by the alternative mechanisms of DNA loss of copy number or hypermethylation. With multiple layers of –omics data available (exposomics, metabolomics, phenomics, proteomics, and transcriptomics et al.), this approach will become more efficient and accurate to help epidemiologists distinguish crucial genes, pathways, epigenetic alterations and environmental factors that indicate cancer risks, development or prognosis. In addition, these biologically functional markers can also be used as preventive or therapeutic targets that enhance the efficacy of cancer control at the population level, as well as in personalized medicine by applying a molecular epidemiological study design integrated with –omics data.

### Availability of supporting data

All supporting data are included as one additional file. The data sets supporting the results of this article are available in the NCBI’s GEO database repository, including GSE54503 (http://www.ncbi.nlm.nih.gov/geo/query/acc.cgi?acc=GSE54503), GSE37988 (http://www.ncbi.nlm.nih.gov/geo/query/acc.cgi?acc=GSE37988) and GSE54751(http://www.ncbi.nlm.nih.gov/geo/query/acc.cgi?acc=GSE54751).

## Additional file

Additional file 1:
**Laboratory methods.** To display details of methods for DNA extraction; the Infinium Methylation 27K/450K assays; TaqMan Low Density Arrays; qRT-PCR and targeted bis-seq. **Figure S1.** DNA methylation results of targeted bis-seq for *GRASP* (covering 25 CpG sites) and *TSPYL5* (covering 57 CpG sites). The heatmaps for *GRASP* and *TSPYL5* contain data for 23 paired non-tumor/tumor samples. One paired sample did not have data for the non-tumor so the data was removed. The non-tumor samples are grouped at the top and the corresponding tumor samples are at the bottom. The far left columns contain the class (non-tumor (N) or tumor (T) and then the patient number. The next columns show the percent methylation for each of CpG site covered and colored according to the chart. The column to the far right contains an average percent methylation for all the CpGs covered for that sample. Overall, within each sample, DNA methylation levels across different CpG sites were consistent no matter tumor tissue status. Most tumor tissues displayed higher levels of DNA methylation compared to relevant adjacent non-tumor tissues for individual CpG site and the mean of all CpG sites. **Figure S2.** DNA methylation comparisons for *GRASP* and *TSPYL5* by targeted bis-seq and 450K approaches. Box-plot diagrams were analyzed to compare the quantities of methylation differences using two different approaches. The two examples of DNA methylation comparisons for *GRASP* and *TSPYL5* indicate that statistically significant DNA hypermethylation was observed in HCC tumor tissue compared to non-tumor tissue. **Figure S3.** Gains and losses of CNV in HCC tumor, precursor and normal liver tissues for six representative genes based on the Oncomine database. Box-plot diagrams were analyzed to compare hypermethylated genes (*CLCN1*, *GRASP* and *TSPYL5*) under-expressed in HCC tumor tissue, and that display no significant losses of CNV compared to precursor and normal liver tissues. In contrast, statistically significant CNV losses were found in HCC tumor tissue for hypermethylated *CDKL2* and *ZNF397OS*, and significant CNV gains were observed for hypomethylated *KCNQ2* and *PTPRN2*, suggesting potential important role of CNVs in regulation these genes’ function. **Figure S4.** Distribution of *GRASP* and *TSPYL5* expression levels for each sample in 24 pairs and 42 pairs of HCC tissues. A consistent and statistically significant under-expression pattern was observed in HCC tumor tissues for *GRASP* and *TSPYL5* in both the 24 and 42 paired samples. **Figure S5.** DNA methylation changes and relevant genes’ (*GRASP*, *TSPYL5*) expression (Log 2 fold change) pattern in discovery (24 pairs) and validation (42 pairs) sets. DNA methylation changes (hyper-, hypo-) and relevant genes’ expression (under-, over-) between individual tumor and adjacent non-tumor tissues were analyzed. There were, respectively 65 and 64 HCC tumor tissues that showed significant DNA hypermethylation for *GRASP* and *TSPYL5*. Highly consistent hypermethylation and repression patterns were observed in tumor tissues for *GRASP* (17/24, 71%) and *TSPYL5* (16/24, 67%) in the training set (Figure S5A). Validation in an additional testing set (Figure S5B) found a similar proportion of tumor tissues with hypermethylation and under-expression pattern for *GRASP* (28/41, 68%) and *TSPYL5* (31/40, 78%). However, less than one third of the tumor tissues showed a heterogeneous pattern of DNA hypermethylation and mRNA over-expression (31% and 27% for *GRASP* and *TSPYL5*, respectively), indicating other potential mechanisms may be involved in the regulation of expression. **Table S1.** Genomic locations of candidate genes and covered CpG sites of amplicons for targeted bis-seq approach. **Table S2.** The clinical and pathological characteristics of 24 HCC patients in the current study. **Table S3.** Oncomine databases for integrative gene expression and copy number variations (CNVs) analyses in liver tissues. **Table S4.** Representative Oncomine mRNAs expression data (Log2) to display concordant patterns with DNA methylation alterations. **Table S5.** Types of miRNAs that target six hypermethylated genes without losses of CNVs. **Table S6.** Comparison of significant expressed miRNAs that target six hypermethylated genes without losses of CNVs. **Table S7.** Frequencies of copy number loss in HCC tumor by methylation status.

## Abbreviations

HCC: Hepatocellular carcinoma
TSGs: Tumor suppressor genes
*APC*: Adenomatous polyposis coli
*p16/CDKN2A*: Cyclin-dependent kinase inhibitor 2A
*RASSF1*: Ras association domain family member 1
CNVs: Copy number variations
miRNAs: microRNAs
Bis-seq: Bisulfite sequencing
GRASP: General receptor for phosphoinositides 1-associated scaffold protein
TSPYL5: Testis-specific protein Y-encoded-like 5
ENCODE: Encyclopedia of DNA Elements
DNase I: Deoxyribonuclease
TNF-α: Tumor necrosis factor alpha
qRT-PCR: Quantitative reverse transcription PCR
TLDA: TaqMan Low Density Arrays
GEO: Gene Expression Omnibus
SMD: Stanford Microarray Database
SD: Standard deviation
H3K4me1: Histone 3 lysine 4 monomethylation
H3K4me3: Histone 3 lysine 4 trimethylation
H3K27ac: Histone 3 lysine 27 acetylation
H3K27me3: Histone 3 lysine 27 trimethylation
