# Supplementary material for: Integrative epigenomic and genomic filtering for methylation markers in hepatocellular carcinomas
Source: BMC Med Genomics. 2015 Jun 10;8:28. doi: 10.1186/s12920-015-0105-1 (PMC4460673; doi:10.1186/s12920-015-0105-1)
Supplement: Additional file 1: — Laboratory methods. To display details of methods for DNA extraction; the Infinium Methylation 27K/450K assays; TaqMan Low Density Arrays; qRT-PCR and targeted bis-seq. Figure S1. DNA methylation results of targeted bis-seq for GRASP (covering 25 CpG sites) and TSPYL5 (covering 57 CpG sites). The heatmaps for GRASP and TSPYL5 contain data for 23 paired non-tumor/tumor samples. One paired sample did not have data for the non-tumor so the data was removed. The non-tumor samples are grouped at the top and the corresponding tumor samples are at the bottom. The far left columns contain the class (non-tumor (N) or tumor (T) and then the patient number. The next columns show the percent methylation for each of CpG site covered and colored according to the chart. The column to the far right contains an average percent methylation for all the CpGs covered for that sample. Overall, within each sample, DNA methylation levels across different CpG sites were consistent no matter tumor tissue status. Most tumor tissues displayed higher levels of DNA methylation compared to relevant adjacent non-tumor tissues for individual CpG site and the mean of all CpG sites. Figure S2. DNA methylation comparisons for GRASP and TSPYL5 by targeted bis-seq and 450K approaches. Box-plot diagrams were analyzed to compare the quantities of methylation differences using two different approaches. The two examples of DNA methylation comparisons for GRASP and TSPYL5 indicate that statistically significant DNA hypermethylation was observed in HCC tumor tissue compared to non-tumor tissue. Figure S3. Gains and losses of CNV in HCC tumor, precursor and normal liver tissues for six representative genes based on the Oncomine database. Box-plot diagrams were analyzed to compare hypermethylated genes (CLCN1, GRASP and TSPYL5) under-expressed in HCC tumor tissue, and that display no significant losses of CNV compared to precursor and normal liver tissues. In contrast, statistically significant CNV losses were [file 12920_2015_105_MOESM1_ESM.pdf]

**Additional file**

# Integrative epigenomic and genomic filtering for methylation markers in hepatocellular carcinomas

Jing Shen[1,*], Clare LeFave[2], Iryna Sirosh[1], Abby B. Siegel[3], Benjamin Tycko[2,4], Regina M. Santella[1]

[1] Department of Environmental Health Sciences, Mailman School of Public Health, Columbia University Medical Center, New York, NY 10032, USA

[2] Institute for Cancer Genetics, Herbert Irving Comprehensive Cancer Center, Columbia University Medical Center, New York, NY 10032, USA

[3] Department of Medicine, Columbia University Medical Center, New York, NY 10032, USA;

[4] Department of Pathology and Cell Biology, Columbia University College of Physicians and Surgeons, New York, NY 10032, USA

* Corresponding author

Email addresses:

JS: js2182@cumc.columbia.edu

CL: clarelefavephd@gmail.com

IS: is2043@cumc.columbia.edu

ABS: aas54@columbia.edu

BT: tycko@icg.cpmc.columbia.edu

RMS: rps1@cumc.columbia.edu

**Laboratory methods**

DNA was extracted from frozen HCC tumor/adjacent non-tumor tissues by standard proteinase K/RNase treatment and phenol/chloroform extraction. The Infinium Methylation 27K/450K assays had been performed according to Illumina's standard protocol [1,2]. The complete methylation profiles have been deposited in NCBI's Gene Expression Omnibus (GEO) [3] and are available through series accession numbers GSE37988 and GSE54503.

Bisulfite treatment was performed on 1μg DNA using the Epitect kit (Qiagen) as per the manufacturer's instructions for CpG methylation sequencing to screen methylation levels of candidate genes. The genomic locations of candidate genes and covered CpG sites of amplicons for targeted bis-seq are given in Supplementary Table 1. Oligonucleotide primers were designed around the CpGs of interest to amplify 200-500 nucleotide amplicons using MethPrimer (http://www.urogene.org/cgi-bin/methprimer/methprimer.cgi) [4]. Primers were synthesized (IDTDNA) and tested on control bisulfite converted DNA. Verification of the amplicon size was done on an agarose gel stained with ethidium bromide. Before synthesis, the tags (CS1 and CS2) were added to the 5' ends of the forward and reverse primers, respectively. These tags were then used to add the sequencing adapter and barcodes (CS1: ACACTGACGACAT GGTTCTACA; CS2: TACGGTAGCAGAGACTTGGTCT). The Access Array Platform from Fluidigm was used to amplify regions of interest with the tested primers. The platform allows for 48 samples and 48 primer pairs with 2,304 individual PCR reactions on the Access Array chip. The PCR was performed with the KAPA HiFi 2x Uracil + polymerase and reaction buffers. The PCR annealing temperature was determined experimentally. After Fluidigm Access Array PCR, the instrument pools all 48 amplicons for each individual sample. Oligonucleotide primers (Fluidigm) designed to contain an Illumina sequencing adapter, a 10 nucleotide barcode and tags complementary to CS1 and CS2 are used for PCR to make the sequencing library of the previously amplified amplicons. Each sample barcode is unique and is used to parse the samples after sequencing. The PCR was performed using a 1:100 dilution of the template from the previous Access Array PCR. The PCR was performed using the Faststart Hi Fidelity kit (Roche) and 14 cycles of PCR. To check for size distribution and for adapter dimmers, the products from both the Access Array PCR and the Barcoding and sequence adapter PCR were run on agarose gels. Verification of a band shift around 59 nucleotides was used to determine whether PCR was successful. The final barcoded libraries were then pooled in equimolar amounts. To remove

any primer dimers, the final pooled library was then cleaned-up using the Agencourt AMPure XP (Beckman Coulter) magnetic beads. Before sample loading on the MiSeq, the library was quantified using the Kapa Library Quantification Kit (Kapabiosystems). Samples were then pooled with 30-50% PhiX (Illumina) and loaded onto the MiSeq (Illumina) for sequencing. Libraries are clustered and sequenced with 250 nucleotide paired-end. The Fastq files generated by sequencing were trimmed for both adapters and for a quality cut off of 30 using Trim Galore (http://www.bioinformatics.babraham.ac.uk/projects/trim_galore/). Sequencing alignment and methylation calls were done via Bismark [5] and bowtie2 [6].

For genome-wide miRNA expression profiles, the RT reaction was carried out by using TaqMan miRNA RT kits with an input of 750ng RNA per sample. TaqMan Low Density Arrays (TLDA, Applied Biosystems, Foster City, CA), covering 670 unique human mature miRNAs were used to generate genome-wide miRNA profiles. Cycle threshold ($C_t$) values were calculated using the SDS2.2.2. U6 snRNA which is stable in liver tissue was used as an endogenous control to normalize the relative expression of miRNAs by the $2^{(-\Delta\Delta Ct)}$ approach [7]. All qRT-PCR reactions were carried out in an ABI 7900HT Fast Real Time PCR System. The TLDA data has been deposited in NCBI's GEO [3] and are available through series accession number GSE54751.

**Supplemental Figure Legend**

**Supplementary Figure S1 DNA methylation results of targeted bis-seq for *GRASP* (covering 25 CpG sites) and *TSPYL5* (covering 57 CpG sites)**

The heatmaps for *GRASP* and *TSPYL5* contain data for 23 paired non-tumor/tumor samples. One paired sample did not have data for the non-tumor so the data was removed. The non-tumor samples are grouped at the top and the corresponding tumor samples are at the bottom. The far left columns contain the class (non-tumor (N) or tumor (T) and then the patient number. The next columns show the percent methylation for each of CpG site covered and colored according to the chart. The column to the far right contains an average percent methylation for all the CpGs covered for that sample.

For *GRASP*, the average number of reads per CpG site was 404 with a minimum of 198 and maximum of 1247 reads. Methylation calling was done by Bismark methylation extractor. The specific CpG locations for 25 CpG sites (numbered 1-25) are: 52401117, 52401133, 52401136, 52401140, 52401142, 52401164, 52401169, 52401181, 52401189, 52401191, 52401198, 52401206, 52401213, 52401222, 52401233, 52401235, 52401245, 52401261, 52401263, 52401274, 52401324, 52401355, 52401363, 52401397 and 52401401.

For *TSPYL5*, the average number of reads per CpG site was 457 with a minimum of 128 and maximum of 3033 reads. Methylation calling was done by Bismark methylation extractor. The specific CpG locations for 57 CpG sites (numbered 1-57) are: 98289964, 98289969, 98289971, 98289973, 98289978, 98289981, 98289985, 98289995, 98290001, 98290011, 98290013, 98290025, 98290035, 98290037, 98290049, 98290055, 98290061, 98290065, 98290077, 98290080, 98290100, 98290113, 98290123, 98290133, 98290137, 98290143, 98290147, 98290159, 98290166, 98290169, 98290172, 98290177, 98290181, 98290194, 98290197, 98290199, 98290207, 98290214, 98290218, 98290228, 98290231, 98290234, 98290239, 98290245, 98290254, 98290261, 98290263, 98290267, 98290284, 98290286, 98290288, 98290294, 98290306, 98290309, 98290326, 98290330 and 98290332.

Overall, within each sample, DNA methylation levels across different CpG sites were consistent no matter tumor tissue status. Most tumor tissues displayed higher levels of DNA methylation compared to relevant adjacent non-tumor tissues for individual CpG site and the mean of all CpG sites.

**Supplementary Figure S2 DNA methylation comparisons for *GRASP* and *TSPYL5* by targeted bis-seq and 450K approaches**

Box-plot diagrams were analyzed to compare the quantities of methylation differences using two different approaches. The two examples of DNA methylation comparisons for *GRASP* and *TSPYL5* indicate that statistically significant DNA hypermethylation was observed in HCC tumor tissue compared to non-tumor tissue.

**Supplementary Figure S3 Gains and losses of CNV in HCC tumor, precursor and normal liver tissues for six representative genes based on the Oncomine database**

Box-plot diagrams were analyzed to compare hypermethylated genes (*CLCN1*, *GRASP* and *TSPYL5*) under-expressed in HCC tumor tissue, and that display no significant losses of CNV compared to precursor and normal liver tissues. In contrast, statistically significant CNV losses were found in HCC tumor tissue for hypermethylated *CDKL2* and *ZNF397OS*, and significant CNV gains were observed for hypomethylated *KCNQ2* and *PTPRN2*, suggesting potential important role of CNVs in regulation these genes' function.

**Supplementary Figure S4 Distribution of *GRASP* and *TSPYL5* expression levels for each sample in 24 pairs and 42 pairs of HCC tissues**

A consistent and statistically significant under-expression pattern was observed in HCC tumor tissues for *GRASP* and *TSPYL5* in both the 24 and 42 paired samples

**Supplementary Figure S5 DNA methylation changes and relevant genes' (*GRASP*, *TSPYL5*) expression (Log 2 fold change) pattern in discovery (24 pairs) and validation (42 pairs) sets**

DNA methylation changes (hyper-, hypo-) and relevant genes' expression (under-, over-) between individual tumor and adjacent non-tumor tissues were analyzed. There were, respectively 65 and 64 HCC tumor tissues that showed significant DNA hypermethylation for *GRASP* and *TSPYL5*. Highly consistent hypermethylation and repression patterns were observed in tumor tissues for *GRASP* (17/24, 71%) and *TSPYL5* (16/24, 67%) in the training set (Supplementary Figure 5A). Validation in an additional testing set (Supplementary Figure 5B) found a similar proportion of tumor tissues with hypermethylation and under-expression pattern for *GRASP* (28/41, 68%) and *TSPYL5* (31/40, 78%). However, less than one third of the tumor tissues showed a heterogeneous pattern of DNA hypermethylation and mRNA over-expression (31% and 27% for *GRASP* and *TSPYL5*, respectively), indicating other potential mechanisms may be involved in the regulation of expression.

**Supplementary Table S1** Genomic locations of candidate genes and covered CpG sites of amplicons for targeted bis-seq approach

**Supplemental Table S2** The clinical and pathological characteristics of 24 HCC patients in the current study

**Supplementary Table S3** Oncomine databases for integrative gene expression and copy number variations (CNVs) analyses in liver tissues

**Supplementary Table S4** Representative Oncomine mRNAs expression data (Log2) to display concordant patterns with DNA methylation alterations

**Supplementary Table S5** Types of miRNAs that target six hypermethylated genes without losses of CNVs

**Supplementary Table S6** Comparison of significant expressed miRNAs that target six hypermethylated genes without losses of CNVs

**Supplementary Table S7** Frequencies of copy number loss in HCC tumor by methylation status

## Supplementary Figure S1

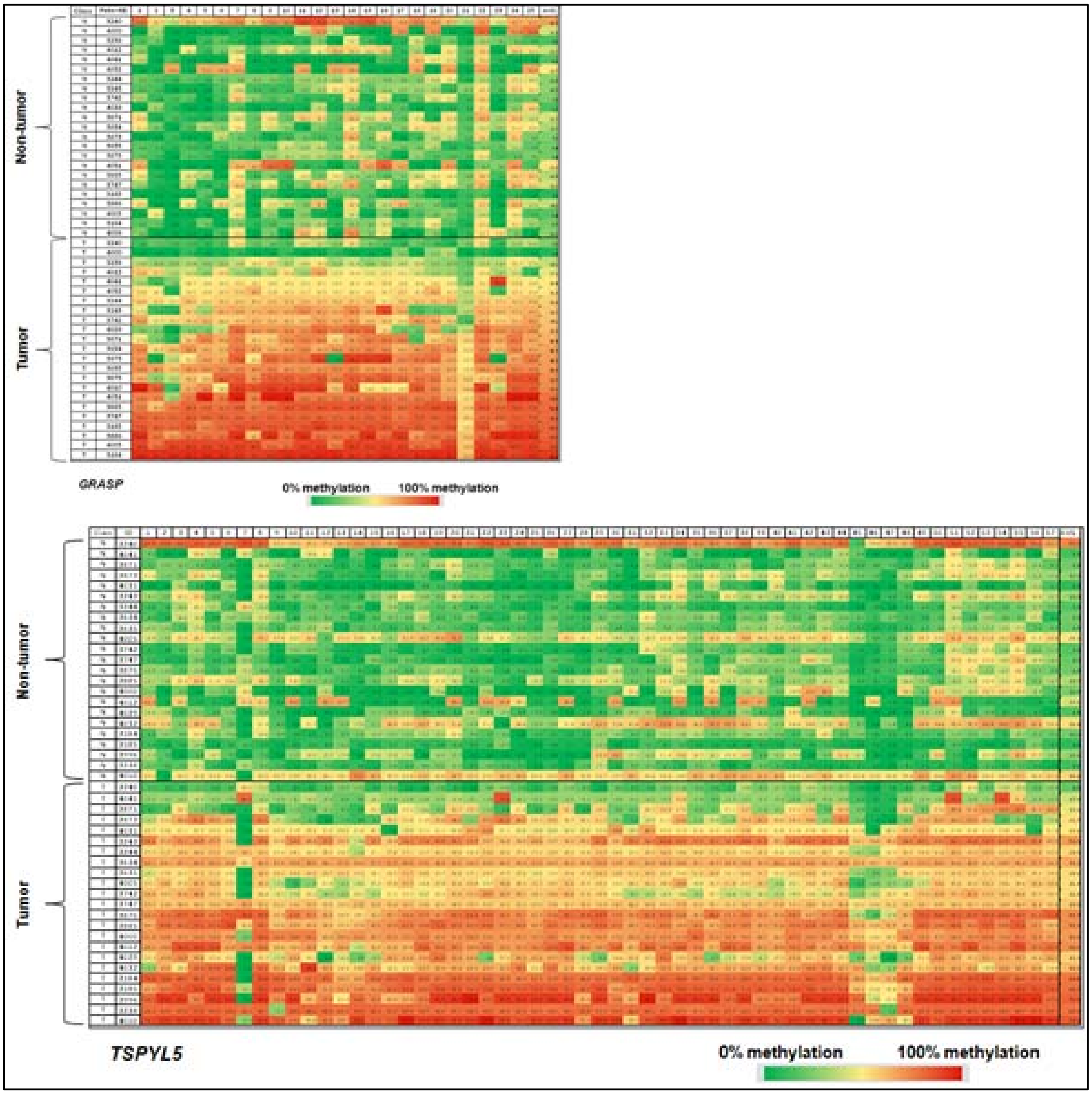

**Supplementary Figure S2**

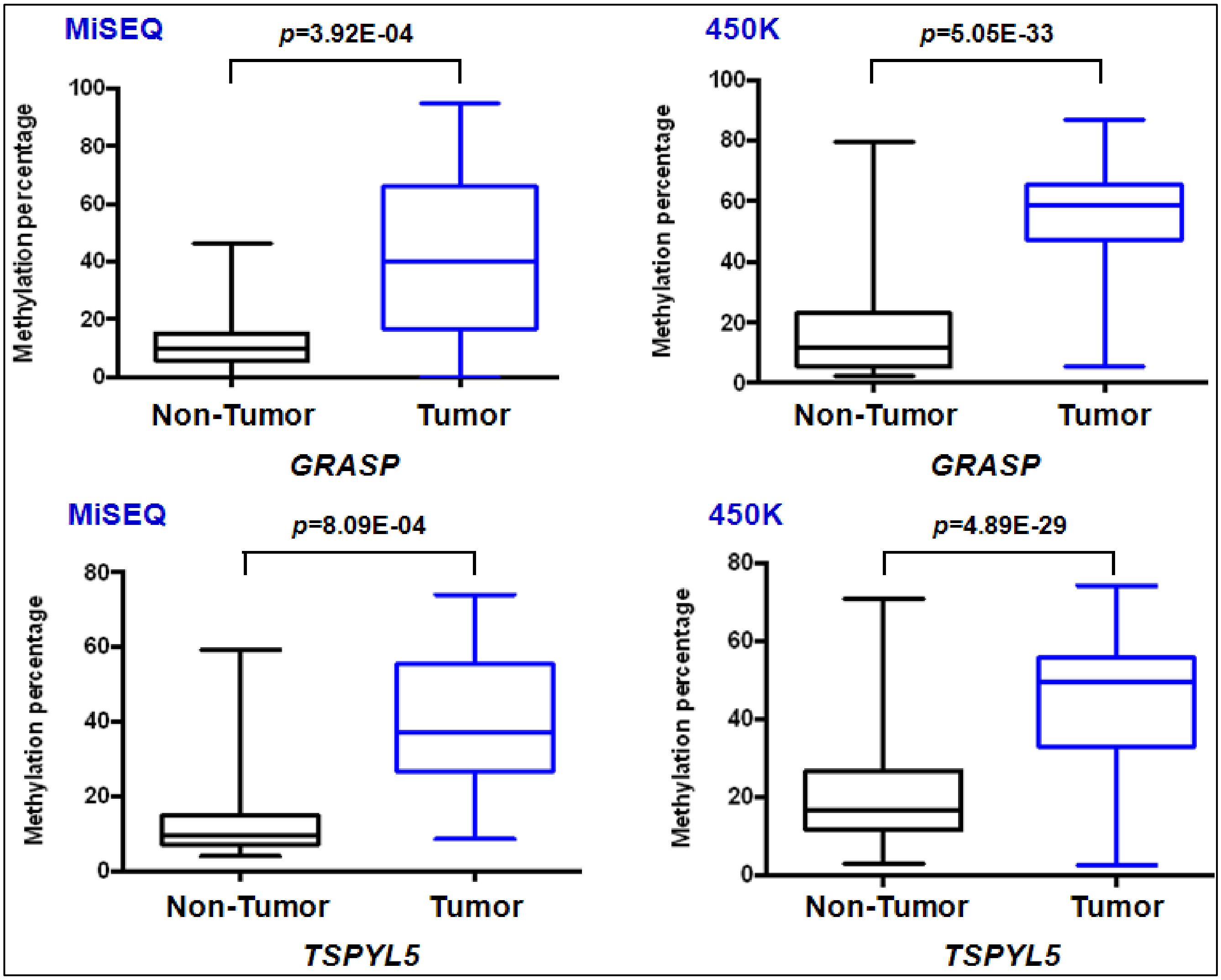

**Supplementary Figure S3**

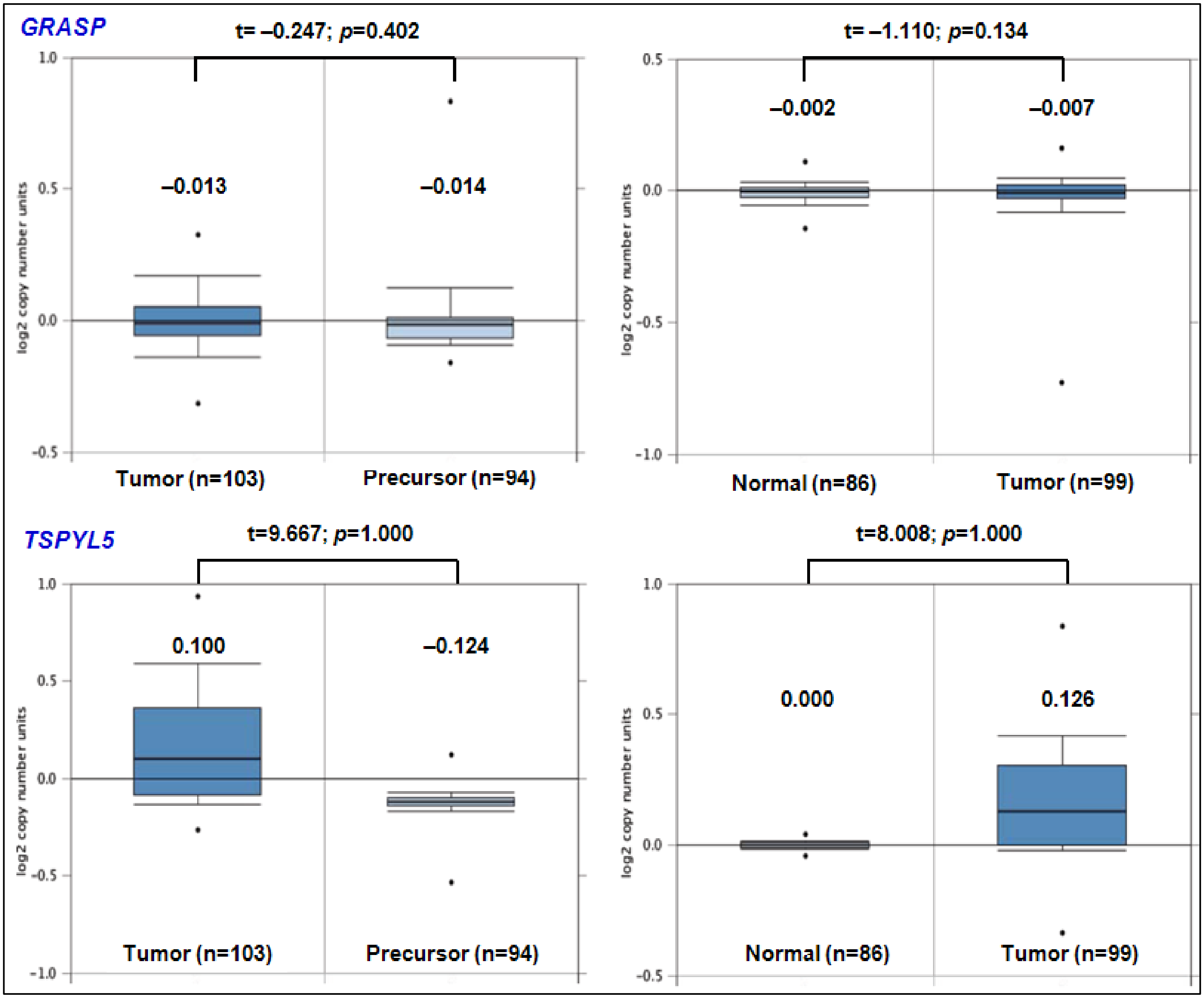

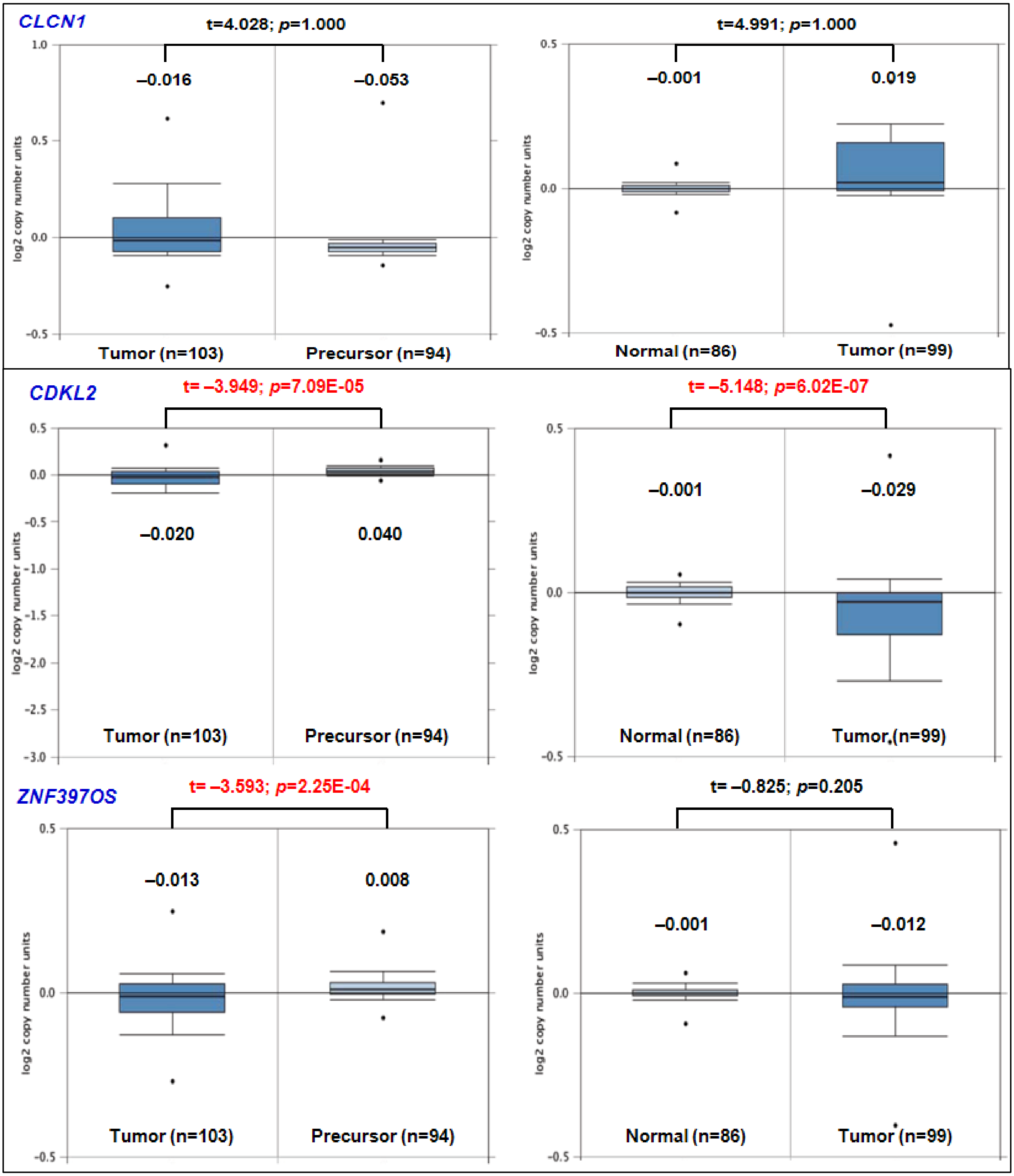

CLCN1
t=4.028; p=1.000
t=4.991; p=1.000
–0.016
–0.053
–0.001
0.019
log2 copy number units
Tumor (n=103)
Precursor (n=94)
Normal (n=86)
Tumor (n=99)
CDKL2
t= –3.949; p=7.09E-05
t= –5.148; p=6.02E-07
–0.020
0.040
–0.001
–0.029
Tumor (n=103)
Precursor (n=94)
Normal (n=86)
Tumor (n=99)
ZNF397OS
t= –3.593; p=2.25E-04
t= –0.825; p=0.205
–0.013
0.008
–0.001
–0.012
Tumor (n=103)
Precursor (n=94)
Normal (n=86)
Tumor (n=99)

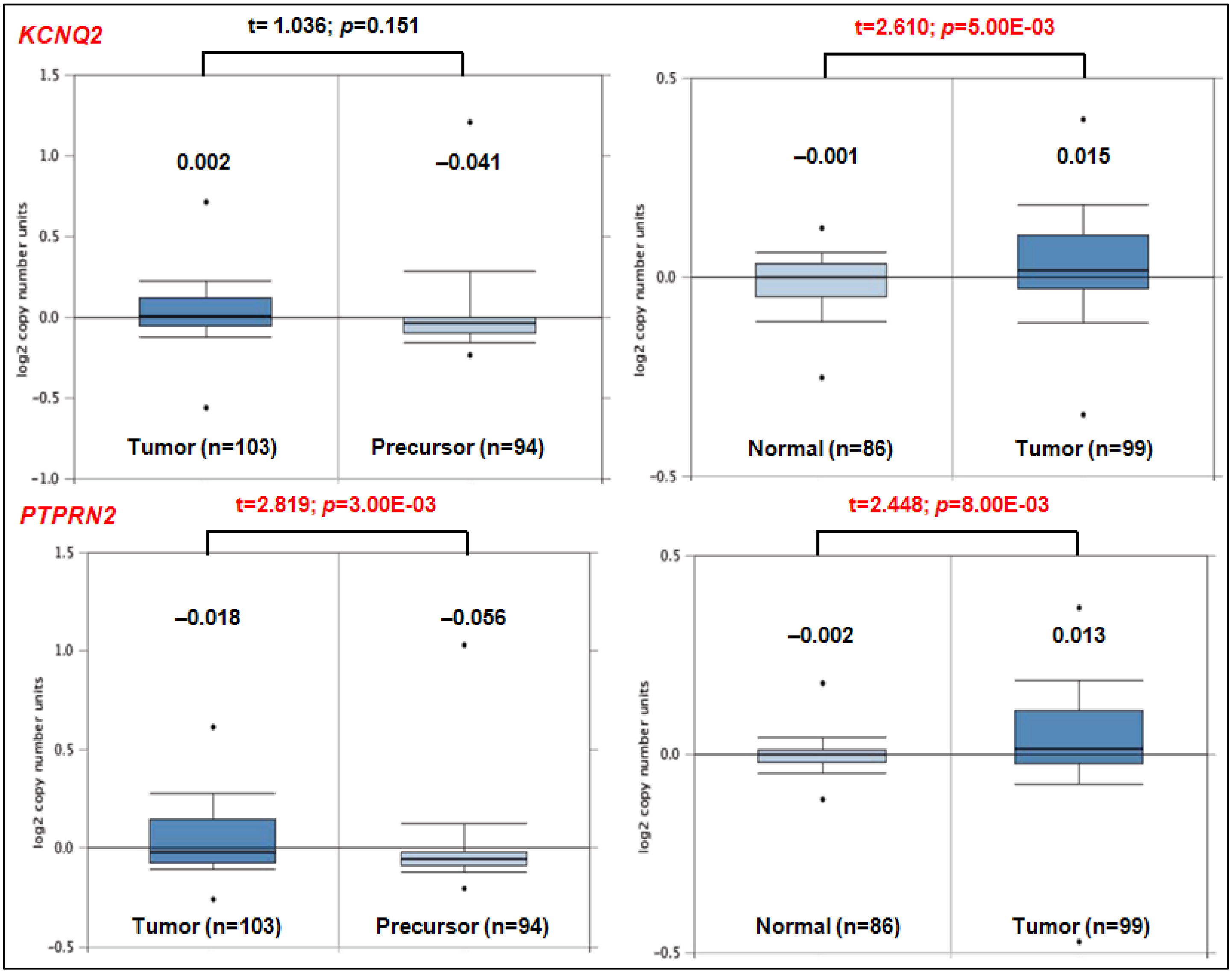
KCNQ2
t= 1.036; p=0.151
t=2.610; p=5.00E-03
0.002
–0.041
–0.001
0.015
log2 copy number units
Tumor (n=103)
Precursor (n=94)
Normal (n=86)
Tumor (n=99)
PTPRN2
t=2.819; p=3.00E-03
t=2.448; p=8.00E-03
–0.018
–0.056
–0.002
0.013
log2 copy number units
Tumor (n=103)
Precursor (n=94)
Normal (n=86)
Tumor (n=99)

**Supplementary Figure S4**

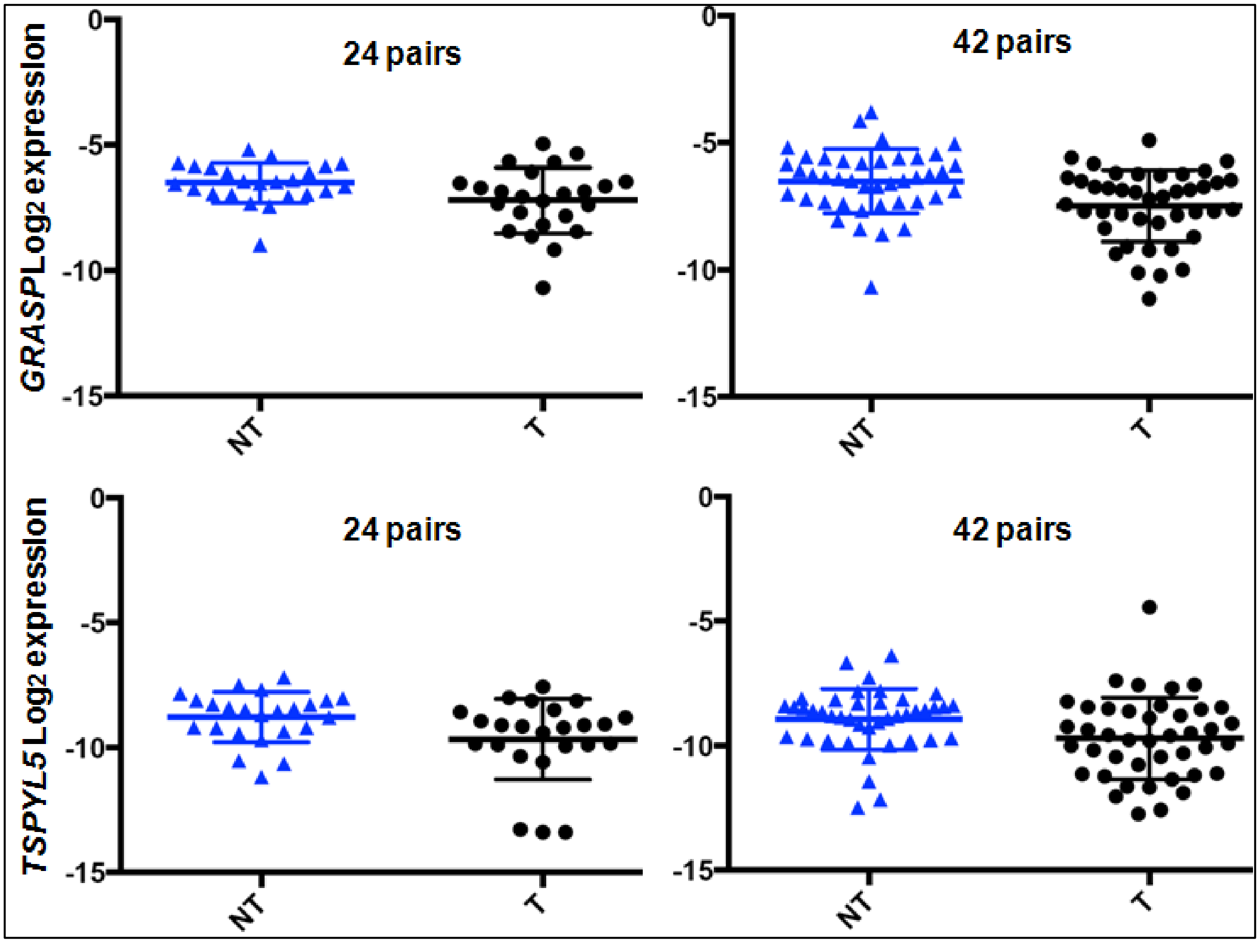

**Supplementary Figure S5**

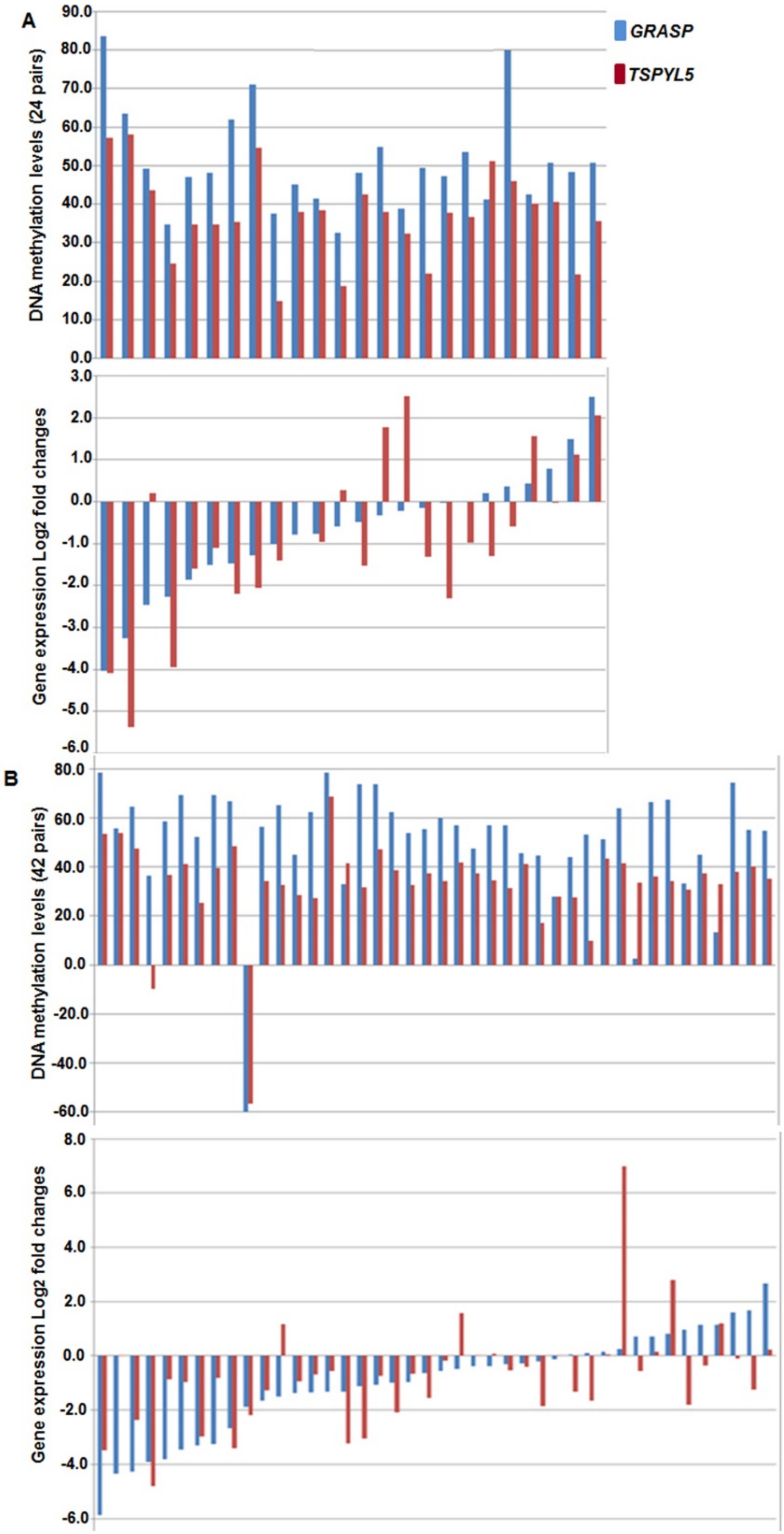

**Supplementary Table S1** Genomic locations of candidate genes and covered CpG sites of amplicons for targeted bisulfite NGS approach

| Gene symbol | Gene name | Gene ID | Starting to ending positions | Chr | Amplicons name | Starting position | Ending position | Amplicon size | No. of measured CpGs |
|---|---|---|---|---|---|---|---|---|---|
| *CDKL2* | cyclin-dependent kinase-like 2 | 8999 | Chr4: 76,555,456-76,556,156 | 4 | CDKL2.a | 76555395 | 76555662 | 268 | 11 |
| *CDKL2* | | | | 4 | CDKL2.b | 76555570 | 76555856 | 287 | 15 |
| *CDKL2* | | | | 4 | CDKL2.c | 76555792 | 76556131 | 340 | 27 |
| *CLCN1* | chloride channel, voltage-sensitive 1 | 1180 | Chr7: 143,042,498-143,043,098 | 7 | CLCN1 | 143042601 | 143043006 | 406 | 28 |
| *DUOX1* | dual oxidase 1 | 53905 | Chr15: 45,421,762-45,422,362 | 15 | DUOX1 | 45421872 | 45422250 | 379 | 36 |
| *FAM66B* | family with sequence similarity 66, member B | 100128890 | Chr8: 7,190,973-7,191,573 | 8 | FAM66B | 7191219 | 7191416 | 198 | 13 |
| *GRASP* | general receptor for phosphoinositides 1-associated scaffold protein | 160622 | Chr12: 52,400,814-52,401,414 | 12 | GRASP.a | 52401097 | 52401426 | 330 | 25 |
| *ILDR2* | immunoglobulin-like domain containing receptor 2 | 387597 | Chr1: 166,916,709-166,917,309 | 1 | ILDR2.a | 166916649 | 166916935 | 287 | 23 |
| *KCNQ2* | potassium voltage-gated channel, KQT-like subfamily, member 2 | 3785 | Chr20: 62,097,381-62,097,981 | 20 | KCNQ2 | 62097446 | 62097902 | 457 | 44 |
| *MAST1* | microtubule associated serine/threonine kinase 1 | 22983 | Chr19: 12,978,206-12,978,806 | 19 | MAST1.a | 12978569 | 12978769 | 201 | 16 |
| *MAST1* | | | | 19 | MAST1.b | 12978207 | 12978458 | 252 | 2 |
| *MAST1* | | | | 19 | MAST1.c | 12978257 | 12978593 | 337 | 19 |
| *NKX6-2* | NK6 homeobox 2 | 84504 | Chr10: 134,598,300-134,599,200 | 10 | NKX6-2.b | 134598414 | 134598676 | 263 | 24 |
| *OTX1* | orthodenticle homeobox 1 | 5013 | Chr2: 63,280,839-63,281,439 | 2 | OTX1.a | 63280940 | 63281111 | 172 | 8 |

| | | | | | | | | | |
|---|---|---|---|---|---|---|---|---|---|
| *OTX1* | | | | 2 | OTX1.b | 63281091 | 63281435 | 345 | 28 |
| *PROKR2* | prokineticin receptor 2 | 128674 | Chr20: 5,282,652-5,283,252 | 20 | PROKR2 | 5282852 | 5283091 | 240 | 14 |
| *PTPRN2* | protein tyrosine phosphatase, receptor type, N polypeptide 2 | 5799 | Chr7:157,932,097-157,932,697 | 7 | PTPRN2 | 157932340 | 157932554 | 215 | 8 |
| *REXO1L2P* | REX1, RNA exonuclease 1 homolog (S. cerevisiae)-like 2 (pseudogene) | 100288527 | Chr8: 86,567,863-86,568,463 | 8 | REXO1L2P | 86568010 | 86568255 | 246 | 12 |
| *SERHL* | serine hydrolase-like | 94009 | Chr22: 42,896,400-42,896,999 | 22 | SERHL.a | 42896583 | 42896788 | 206 | 16 |
| *SERHL* | | | | 22 | SERHL.b | 42896254 | 42896611 | 358 | 11 |
| *SPAG6* | sperm associated antigen 6 | 9576 | Chr10: 22,634,126-22,634,726 | 10 | SPAG6.b | 22634201 | 22634563 | 363 | 53 |
| *SPDYA* | speedy homolog A (Xenopus laevis) | 245711 | Chr2: 29,033,480-29,034,080 | 2 | SPDYA.b | 29034114 | 29034378 | 265 | 11 |
| *TRIL* | TLR4 interactor with leucine-rich repeats | 9865 | Chr7: 28,997,378-28,998,178 | 7 | TRIL.a | 28997312 | 28997564 | 253 | 16 |
| *TRIL* | | | | 7 | TRIL.b | 28997531 | 28997911 | 381 | 22 |
| *TRIL* | | | | 7 | TRIL.c | 28997727 | 28998009 | 283 | 17 |
| *TRIL* | | | | 7 | TRIL.e | 28997899 | 28998338 | 440 | 44 |
| *TSPYL5* | TSPY-like 5 | 85453 | Chr8: 98,289,929-98,290,529 | 8 | TSPYL5 | 98289942 | 98290359 | 418 | 57 |
| *USP44* | ubiquitin specific peptidase 44 | 84101 | Chr12: 95,942,607-95,943,207 | 12 | USP44.c | 95943049 | 95943251 | 203 | 9 |
| *ZNF397OS* | zinc finger and SCAN domain containing 30 | 100101467 | Chr18: 32,847,266-32,847,866 | 18 | ZNF397OS.a | 32847227 | 32847538 | 312 | 32 |

**Supplemental Table S2** The clinical and pathological characteristics of 24 HCC patients in the current study

| Variables | HCC patients |
|---|---|
| Age at diagnosis, Mean ± SD | 57.1 ± 7.5 |
| Age at diagnosis, No (%) | |
| < 60 yrs | 14 (58) |
| ≥ 60 yrs | 10 (42) |
| Gender, No (%) | |
| Male | 20 (83) |
| Female | 4 (17) |
| Ethnicity, No (%) | |
| Caucasian | 13 (54) |
| African-American | 2 (8) |
| Hispanic | 2 (8) |
| Asian | 6 (25) |
| Others | 1 (4) |
| Viral infection, No (%) | |
| HBV (−), HCV (−) | 4 (17) |
| HBV (−), HCV (+) | 8 (33) |
| HBV (+), HCV (−) | 8 (33) |
| HBV (+), HCV (+) | 4 (17) |
| Cigarette smoking, No (%) | |
| Never | 11 (46) |
| Ever | 11 (46) |
| Missing | 2 (8) |
| Alcohol drinking, No (%) | |
| No | 11 (46) |
| Yes | 11 (46) |
| Missing | 2 (8) |
| AFP (ng/mL), Mean ± SD | 831.9 ± 2630.1 |
| Cirrhosis, No (%) | |
| No | 3 (13) |
| Yes | 21 (87) |
| Tumor grade*, No (%) | |
| I-II | 9 (37) |
| III | 12 (50) |
| IV | 3 (13) |
| Treatment, No (%) | |
| Resection alone | 8 (33) |
| Transplantation alone | 14 (59) |
| IV | 2 (8) |
| Survival, No (%) | |
| Yes | 15 (63) |
| No | 9 (37) |

* Edmondson and Steiner grade

**Supplementary Table S3** Oncomine databases for integrative gene expression and copy number variations (CNVs) analyses in liver tissues

| First author | Journal, Year | Sample size | | | Risk factors |
|---|---|---|---|---|---|
| | | NT | Pre | T * | |
| **Gene expression databases** | | | | | |
| Roessler S. | Cancer Res, 2010 | 220 | 0 | 225 | Predominantly HBV-positive |
| Archer KJ. | Cancer Epidemiol Biomarkers Prev, 2009 | 0 | 47 | 16 | 100% HCV positive |
| Mas VR. | Mol Med, 2009 | 19 | 58 | 38 | 100% HCV positive |
| Wurmbach E. | Hepatology, 2007 | 10 | 30 | 35 | 100% HCV positive |
| Chen X. | Mol Biol Cell, 2002 | 76 | 7 | 104 | Predominantly HBV-positive |
| Total | | 325 | 142 | 418 | |
| **CNVs databases** | | | | | |
| Chiang DY. | Cancer Res, 2008 | | 94 | 103 | 100% HCV positive |
| Guichard C. | Nat Genet, 2012 | 86 | | 99 | 36 HBV; 25 HCV, 55 Alcohol † |
| TCGA (The Cancer Genome Atlas) | Unpublished, 2012 | 59 | | 97 | Unknown |
| Total | | 145 | 94 | 209 | |

* NT: Normal tissue; Pre: Precursor tissue; T: HCC tumor tissue

† 16 cases carrying 2 or more risk factors

**Supplementary Table S4** Representative Oncomine mRNAs expression data (Log2) to display concordant patterns with DNA methylation alterations

| Gene symbol | Sample size (NT/Pre/T)* | Normal Median (range) | Precursor Median (range) | Tumor Median (range) | Fold-change (T vs. NT) | t-test (T vs. NT) | Adjusted *P* value (T vs. NT) | Fold-change (T vs. Pre) | t-test (T vs. Pre) | Adjusted *P* value (T vs. Pre) | References |
|---|---|---|---|---|---|---|---|---|---|---|---|
| *CDKL2* | 220/0/225 | -0.986 (-1.369, 0.564) | ND | -1.025 (-1.375, -0.234) | -1.040 | -2.868 | **2.00E-03** | ND | ND | ND | Roessler S. et al. Cancer Res 2010 |
| | 21/0/22 | -0.819 (-1.022, -0.456) | ND | -0.981 (-1.201, -0.755) | -1.119 | -3.891 | **1.83E-04** | ND | ND | ND | Roessler S. et al. Cancer Res 2010 |
| ***CLCN1*** | 10/30/35 | -0.461 (-0.705, 1.144) | -0.631 (-0.885, 0.179) | -0.677 (-0.84, 0.028) | -1.143 | -2.209 | **2.40E-02** | -1.042 | -1.091 | 1.40E-01 | Wurmbach E. et al. Hepatology 2007 |
| | 220/0/225 | -0.615 (-1.149, 0.407) | ND | -0.71 (-1.201, 0.398) | -1.058 | -3.512 | **2.45E-04** | ND | ND | ND | Roessler S. et al. Cancer Res 2010 |
| | 21/0/22 | -0.578 (-0.794, -0.045) | ND | -0.698 (-1.017, -0.098) | -1.112 | -2.525 | **8.00E-03** | ND | ND | ND | Roessler S. et al. Cancer Res 2010 |
| | 0/47/16 | ND | -0.27 (-0.631, 0.405) | -0.416 (-0.894, 0.332) | ND | ND | ND | -1.120 | -1.989 | **3.00E-02** | Archer KJ. et al. Cancer Epidemiol Biomarkers Prev 2009 |
| ***DUOX1*** | 10/30/35 | -0.795 (-0.878, -0.723) | -0.82 (-0.952, -0.505) | -0.838 (-1.075, -0.541) | -1.036 | -2.473 | **1.00E-02** | -1.039 | -2.376 | **1.00E-02** | Wurmbach E. et al. Hepatology 2007 |
| ***GRASP*** | 10/30/35 | 2.134 (1.158, 2.486) | 2.21 (1.791, 2.727) | 1.828 (0.941, 2.526) | -1.242 | -2.426 | **1.40E-02** | -1.394 | -6.187 | **3.69E-08** | Wurmbach E. et al. Hepatology 2007 |
| *MAST1* | 76/7/104 | 0.595 (-1.626, 2.22) | 0.725 (-0.166, 1.304) | 0.35 (-1.255, 1.883) | -1.121 | -2.268 | **1.20E-02** | -1.157 | -1.009 | 1.74E-01 | Chen X. et al. Mol Biol Cell 2002 |
| *NKX6-2* | 10/30/35 | -1.694 (-1.805, -1.167) | -1.71 (-1.806, -1.62) | -1.712 (-1.807, -1.608) | -1.046 | -1.154 | 8.61E-01 | -1.002 | -0.275 | 6.08E-01 | Wurmbach E. et al. Hepatology 2007 |
| *OTX1* | 10/30/35 | -1.786 (-1.906, -1.356) | -1.816 (-1.935, -1.503) | -1.818 (-1.958, -1.267) | -1.015 | -0.362 | 3.61E-01 | 1.031 | 1.312 | 9.02E-01 | Wurmbach E. et al. |

| | | | | | | | | | | | |
|---|---|---|---|---|---|---|---|---|---|---|---|
| *SPAG6* | 10/30/35 | -1.518 (-1.638, -1.457) | -1.594 (-1.693, -1.471) | -1.6 (-1.7, -1.028) | -1.035 | -1.985 | **2.80E-02** | -1.003 | -0.177 | 4.30E-01 | Wurmbach E. et al. Hepatology 2007 |
| | 19/58/38 | -1.532 (-1.661, -1.256) | -1.729 (-2.254, -1.293) | -1.658 (-1.845, -1.393) | -1.095 | -4.293 | **6.03E-05** | 1.033 | 1.398 | 9.17E-01 | Mas VR. et al. Mol Med 2009 |
| | 21/0/22 | -0.998 (-1.318, -0.657) | ND | -1.097 (-1.49, -0.839) | -1.077 | -2.453 | **9.00E-03** | ND | ND | ND | Roessler S. et al. Cancer Res 2010 |
| *SPDYA* | 10/30/35 | -1.607 (-1.703, -1.381) | -1.657 (-1.785, -1.432) | -1.655 (-1.763, -1.242) | -1.036 | -1.629 | 6.20E-02 | 1.000 | 0.021 | 5.08E-01 | Wurmbach E. et al. Hepatology 2007 |
| *TRIL* | 10/30/35 | -0.852 (-0.904, -0.765) | -1.349 (-1.469, -0.631) | -0.831 (-1.203, 1.051) | 1.082 | 1.858 | 9.64E-01 | 1.128 | 1.839 | 9.64E-01 | Wurmbach E. et al. Hepatology 2007 |
| | 19/58/38 | -0.811 (-1.131, -0.521) | -0.972 (-1.556, -0.524) | -0.969 (-1.445, -0.362) | 1.107 | -3.028 | **2.00E-03** | 1.023 | 0.735 | 7.68E-01 | Mas VR. et al. Mol Med 2009 |
| ***TSPYL5*** | 10/30/35 | 1.527 (0.195, 2.195) | 1.585 (-0.079, 3.293) | -0.148 (-1.218, 1.844) | -2.308 | -4.460 | **2.85E-04** | -2.723 | -6.888 | **3.39E-09** | Wurmbach E. et al. Hepatology 2007 |
| *USP44* | 10/30/35 | -1.201 (-1.295, -1.098) | -1.223 (-1.346, -1.075) | -1.22 (-1.544, 0.225) | 1.006 | 0.191 | 5.75E-01 | 1.025 | 0.780 | 7.80E-01 | Wurmbach E. et al. Hepatology 2007 |
| | 76/7/104 | -0.014 (-3.269, 0.913) | 0.071 (-0.353, 0.607) | 0.183 (-1.588, 1.508) | 1.215 | 3.265 | 9.99E-01 | 1.042 | 0.454 | 6.70E-01 | Chen X. et al. Mol Biol Cell 2002 |
| *ZNF397OS* | 10/30/35 | -1.212 (-1.179, -0.885) | 0.592 (0.323, 1.17) | -1.17 (-1.329, -0.467) | -1.228 | -0.756 | 2.28E-01 | 1.020 | 0.306 | 6.20E-01 | Wurmbach E. et al. Hepatology 2007 |
| | 76/7/104 | -0.196 (-1.362, 3.632) | 0.373 (-0.114, 1.274) | 0.002 (-2.652, 2.748) | -1.058 | -0.598 | 2.75E-01 | -1.358 | -2.380 | **2.20E-02** | Chen X. et al. Mol Biol Cell 2002 |
| *KCNQ2* | 10/30/35 | -1.929 (-2.051, -1.671) | -1.936 (-2.119, -1.583) | -1.934 (-2.106, -0.714) | 1.028 | 0.696 | 2.45E-01 | 1.022 | 0.632 | 2.65E-01 | Wurmbach E. et al. Hepatology 2007 |
| | 76/7/104 | -2.191 (-4.735, | -1.281 (-2.885, - | -1.281 (-2.796, - | 1.273 | 0.624 | 2.94E-01 | -1.448 | -1.467 | 9.10E-01 | Chen X. et al. |

| | | | | | | | | | | | |
|---|---|---|---|---|---|---|---|---|---|---|---|
| | | 0.974) | 0.491) | 1.099) | | | | | | | Mol Biol Cell 2002 |
| | 19/58/38 | -1.091 (-1.231, -0.832) | -1.65 (-2.203, -1.289) | -1.057 (-1.431, -0.735) | 1.024 | 0.947 | 1.74E-01 | 1.110 | 4.531 | **9.06E-06** | Mas VR. et al. Mol Med 2009 |
| *PTPRN2* | 10/30/35 | -0.387 (-0.473, 0.064) | -0.404 (-0.931, 1.918) | -0.439 (-1.406, 0.699) | -1.076 | -1.579 | 9.37E-01 | -1.225 | -2.701 | 9.95E-01 | Wurmbach E. et al. Hepatology 2007 |
| | 19/58/38 | -0.127 (-0.526, 0.502) | 0.223 (-0.636, 0.88) | 0.17 (-0.534, 1.997) | 1.280 | 3.578 | **3.67E-04** | 1.005 | 0.159 | 4.37E-01 | Mas VR. et al. Mol Med 2009 |
| *APC* | 19/58/38 | -1.737 (-1.979, -1.481) | -1.694 (-2.061, -1.184) | -1.881 (-2.167, -1.651) | -1.109 | -3.925 | **2.20E-04** | -1.139 | -6.193 | **7.80E-09** | Mas VR. et al. Mol Med 2009 |
| *P16* | 220/0/225 | -0.289 (-0.996, 1.197) | ND | -0.549 (-1.375, 0.983) | -1.243 | -8.860 | **1.10E-17** | ND | ND | ND | Roessler S. et al. Cancer Res 2010 |
| | 19/58/38 | -0.324 (-0.478, -0.154) | -0.274 (-0.561, 0.482) | -0.333 (-0.804, 0.149) | 1.004 | 0.192 | 5.76E-01 | -1.066 | -2.431 | **9.00E-03** | Mas VR. et al. Mol Med 2009 |

* NT: Normal tissue; Pre: Precursor tissue; T: HCC tumor tissue

ND: No data

**Supplementary Table S5** Types of miRNAs target six hypermethylated genes without losses of CNVs

| Target gene symbol | No. of miRNAs targeting this gene | No. of detectable miRNAs | Detectable miRNAs | No. of significant miRNAs | Significant miRNAs | Regulation in HCC tumor |
|---|---|---|---|---|---|---|
| *CLCN1* | 15 | 8 | hsa-let-7b; hsa-let-7e; hsa-miR-149*; hsa-miR-601; hsa-miR-638; hsa-miR-642; hsa-miR-671; hsa-miR-95 | 2 | hsa-let-7b; hsa-let-7e | Down |
| *GRASP* | 20 | 8 | hsa-miR-331-3p; hsa-miR-132; hsa-miR-212; hsa-miR-622; hsa-miR-320b; hsa-miR-582-5p; hsa-miR-9*; hsa-let-7f-2* | 1 | hsa-miR-320b | Down |
| *MAST1* | 4 | 3 | hsa-miR-493*; hsa-let-7g*; hsa-let-7a | 1 | hsa-let-7a | Down |
| *SPAG6* | 7 | 1 | hsa-miR-888 | 0 | ND | ND |
| *TRIL* | 51 | 27 | hsa-miR-429; hsa-miR-411; hsa-miR-29a; hsa-miR-128; hsa-miR-30d; hsa-miR-30a; hsa-miR-629; hsa-miR-489; hsa-miR-378; hsa-miR-23a; hsa-miR-200b; hsa-miR-497*; hsa-miR-130a*; hsa-miR-27a; hsa-miR-422a; hsa-miR-200c; miR-23b; hsa-miR-30c; hsa-miR-125a-5p; hsa-miR-29c; hsa-miR-944; hsa-miR-125b; hsa-miR-30b; hsa-miR-543; hsa-miR-27b; hsa-miR-30e-3p; hsa-miR-29b | 9 | hsa-miR-30a; hsa-miR-378; hsa-miR-497*; hsa-miR-130a*; hsa-miR-422a; hsa-miR-29c; hsa-miR-125b; hsa-miR-27b; hsa-miR-30e-3p | Down |
| *TSPYL5* | 35 | 18 | hsa-miR-193a-3p; hsa-let-7d; hsa-miR-29b-1*; hsa-let-7g; hsa-let-7a; hsa-miR-193b; hsa-miR-98; hsa-miR-186*; hsa-miR-27a; hsa-miR-1305; hsa-let-7e; hsa-miR-150; hsa-let-7c; hsa-miR-224; hsa-let-7b; hsa-miR-320b; hsa-let-7f; hsa-miR-27b | 8 | hsa-let-7a; hsa-let-7eb; hsa-let-7c; hsa-let-7e; hsa-let-7g; hsa-miR-320b; hsa-miR-193a-3p; hsa-miR-27b | Down |

ND: No data

**Supplementary Table S6** Comparison of significant expressed miRNAs that target six hypermethylated genes without losses of CNVs

| Target gene symbol | Name of significant miRNAs | Non-tumor Mean (Log2), SD | Tumor Mean (Log2), SD | Fold change (Log2)* | Adjusted *P* value ** | Regulation in HCC tumor |
|---|---|---|---|---|---|---|
| *CLCN1* | hsa-let-7b | -4.53, 0.86 | -5.89, 1.18 | -1.36 | **8.94E-03** | Down |
| | hsa-let-7e | -4.05, 0.82 | -4.78, 0.63 | -0.73 | **3.86E-02** | Down |
| *GRASP* | hsa-miR-320b | -8.97, 0.75 | -10.10, 0.96 | -1.13 | **8.95E-03** | Down |
| *MAST1* | hsa-let-7a | -6.43, 0.64 | -7.35, 0.86 | -0.92 | **1.42E-02** | Down |
| *SPAG6* | has-miR-888 | -13.19, 1.60 | -13.08, 1.34 | 0.11 | 8.67E-01 | UP |
| *TRIL* | hsa-miR-30a | -3.84, 0.69 | -5.17, 0.86 | -1.33 | **1.28E-03** | Down |
| | hsa-miR-378 | -3.03, 0.84 | -4.68, 1.14 | -1.65 | **1.70E-03** | Down |
| | hsa-miR-497* | -8.77, 0.94 | -10.43, 1.56 | -1.66 | **1.00E-02** | Down |
| | hsa-miR-130a* | -13.72, 1.61 | -16.38, 0.80 | -2.66 | **3.34E-02** | Down |
| | hsa-miR-422a | -7.04, 1.02 | -8.72, 1.42 | -1.68 | **7.15E-03** | Down |
| | hsa-miR-29c | -6.48, 0.51 | -7.28, 1.04 | -0.80 | **4.00E-02** | Down |
| | hsa-miR-125b | -3.44, 0.88 | -4.97, 1.03 | -1.53 | **2.20E-03** | Down |
| | hsa-miR-27b | -5.69, 0.79 | -6.48, 0.70 | -0.79 | **2.84E-02** | Down |
| | hsa-miR-30e-3p | -4.01, 0.58 | -5.12, 0.56 | -1.11 | **3.82E-04** | Down |
| *TSPYL5* | hsa-miR-193a-3p | -8.21, 1.06 | -9.53, 0.88 | -1.32 | **7.58E-03** | Down |
| | hsa-let-7g | -3.43, 0.67 | -4.11, 0.55 | -0.68 | **2.32E-02** | Down |
| | hsa-let-7a | -6.43, 0.64 | -7.35, 0.86 | -0.92 | **1.42E-02** | Down |
| | hsa-let-7e | -4.05, 0.82 | -4.78, 0.63 | -0.73 | **3.86E-02** | Down |
| | hsa-let-7c | -6.88, 0.50 | -8.62, 1.08 | -1.74 | **2.24E-04** | Down |
| | hsa-let-7b | -4.53, 0.86 | -5.89, 1.18 | -1.36 | **8.94E-03** | Down |
| | hsa-miR-320b | -8.97, 0.75 | -10.10, 0.96 | -1.13 | **8.95E-03** | Down |
| | hsa-miR-27b | -5.69, 0.79 | -6.48, 0.70 | -0.79 | **2.84E-02** | Down |

* $Log_2$(Fold change)>0 indicates miRNAs over-expression, <0 indicates miRNAs under-expression in tumor tissue;
** Bonferroni correction for multiple testing

**Supplementary Table S7** Frequencies of copy number loss in HCC tumor by methylation status

| Genes | No. of DNA methylation changes | | No. of copy number loss (%) | *p* value |
|---|---|---|---|---|
| *PISD* | Hyper- | 47 | 1 (2.1) | |
| | Hypo- | 19 | 12 (63.2) | **<.0001** |
| *TXNRD1* | Hyper- | 51 | 6 (11.8) | |
| | Hypo- | 15 | 6 (40.0) | **0.0347** |
| *LOC729991-MEF2B* | Hyper- | 51 | 1 (2.0) | |
| | Hypo- | 15 | 10 (66.7) | **<.0001** |
| *CCNI2* | Hyper- | 51 | 1 (2.0) | |
| | Hypo- | 15 | 9 (60.0) | **<.0001** |
| *ISYNA1* | Hyper- | 52 | 2 (3.8) | |
| | Hypo- | 14 | 6 (42.9) | **0.0005** |
| *CORO2B* | Hyper- | 52 | 3 (5.8) | |
| | Hypo- | 14 | 5 (35.7) | **0.0097** |
| *C17orf46* | Hyper- | 50 | 1 (2.0) | |
| | Hypo- | 16 | 6 (37.5) | **0.0004** |
| *FCHSD1* | Hyper- | 52 | 1 (1.9) | |
| | Hypo- | 14 | 4 (28.6) | **0.0055** |

## References


1. Shen J, Wang S, Zhang YJ, Kappil M, Wu HC, Kibriya MG et al. **Genome-wide DNA methylation profiles in hepatocellular carcinoma**. *Hepatology* 2012,55:1799-1808.

2. Shen J, Wang S, Zhang YJ, Wu HC, Kibriya MG, Jasmine F et al. **Exploring genome-wide DNA methylation profiles altered in hepatocellular carcinoma using Infinium HumanMethylation 450 BeadChips**. *Epigenetics* 2013,8:34-43.

3. Barrett T, Troup DB, Wilhite SE, Ledoux P, Rudnev D, Evangelista C et al. **NCBI GEO: archive for high-throughput functional genomic data**. *Nucleic Acids Res* 2009,37:D885-D890.

4. Li LC, Dahiya R. **MethPrimer: designing primers for methylation PCRs**. *Bioinformatics* 2002,18:1427-1431.

5. Krueger F, Andrews SR. **Bismark: a flexible aligner and methylation caller for Bisulfite-Seq applications**. *Bioinformatics* 2011,27:1571-1572.

6. Langmead B, Salzberg SL. **Fast gapped-read alignment with Bowtie 2**. *Nat Methods* 2012,9:357-359.

7. Schmittgen TD, Livak KJ. **Analyzing real-time PCR data by the comparative C(T) method**. *Nat Protoc* 2008,3:1101-1108.
